# Electronic Microenvironment Regulation of Bismuth‐salophen Single‐site Catalysts for Enhanced Selectivity in CO_2_ Electrolysis to Formic Acid

**DOI:** 10.1002/advs.202502061

**Published:** 2025-06-10

**Authors:** Tianxing Wang, Tian (Leo) Jin, Zhiping Liu, Jingtao Wang, Yue Gong, Ming Ma, Jie Chen, Shaohua Shen, Rongqian Wu, Yu‐Cheng Huang, Ying Rui Lu, Yi Lyu, Xiaofei Liu

**Affiliations:** ^1^ Department of Hepatobiliary Surgery First Affiliated Hospital of Xi'an Jiaotong University Xi'an 710049 China; ^2^ Center for Regenerative and Reconstructive Medicine Med‐X Institute the First Affiliated Hospital of Xi'an Jiaotong University Xi'an 710049 China; ^3^ School of Future Technology Xi'an Jiaotong University Xi'an 710049 China; ^4^ Department of Applied Chemistry School of Chemistry MOE Key Laboratory for Nonequilibrium Synthesis and Modulation of Condensed Matter Xi'an Key Laboratory of Sustainable Energy Materials Chemistry State Key Laboratory of Electrical Insulation and Power Equipment State Key Laboratory for Mechanical Behavior of Materials Xi'an Jiaotong University Xi'an 710049 China; ^5^ CAS Key Laboratory of Standardization and Measurement for Nanotechnology CAS Center for Excellence in Nanoscience National Center for Nanoscience and Technology Beijing 100190 China; ^6^ School of Chemical Engineering and Technology Xi'an Jiaotong University Xi'an 710049 China; ^7^ International Research Center for Renewable Energy State Key Laboratory of Multiphase Flow in Power Engineering Xi'an Jiaotong University Xi'an 710049 China; ^8^ National Synchrotron Radiation Research Center Hsinchu Taiwan 300092

**Keywords:** bismuth salophen, Co_2_ Reduction reaction, electronic perturbation, single‐site catalysts

## Abstract

Electrocatalytic CO_2_ reduction reactions (CO_2_RR) hold significant industrial potential, with Bi‐based catalysts demonstrating notable advantages in promoting the formation of formic acid (HCOOH). Despite progress, developing selective and efficient catalysts for CO_2_RR remains challenging. In this study, Bi single‐site catalysts (SSCs) is designed modified with various electronegative groups (─F, ─H, and ─OMe (─OCH_3_)) to improve the CO_2_RR selectivity of Bi through atomic‐level microenvironment tuning. Among these, ─F group‐modulated Bi‐Sal‐F catalyst exhibited a high Faradaic efficiency (FE) of 95% for HCOOH within the current density range of −0.1 to −0.5 A cm^−2^. In contrast, the microenvironment modification with the neutral ─H group and the electron‐donating ─OMe group both led to decreased HCOOH selectivity and promoted HER. Combined with theoretical calculations, this is revealed that the microenvironment regulated by the ─F group strongly correlates with the catalytic activity of the metal center. By reducing the band gap and electron density of Bi, this microenvironment enhances the Bi center's ability to adsorb CO_2_ via oxygen‐coordinated, while effectively lowering the activation barrier for intermediate formation during CO_2_RR, thus promoting high selectivity for HCOOH production. This finding provides valuable theoretical insights and holds great potential for the design of highly efficient SSCs with microenvironment control.

## Graphical abstract

The microenvironment modulation of Bi SSCs via electronegative group (─F/─H/─OMe) modification demonstrably suppresses the HER at high current densities, achieving a HCOOH FE of 95%. The ─F group optimizes the electronic structure of Bi, significantly enhancing CO2 adsorption and reducing the energy barrier for *OCHO intermediate formation, thereby enabling efficient CO_2_RR to HCOOH.

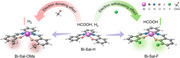

## 1 Introduction

The electrochemical CO_2_ reduction reaction (CO_2_RR) driven by renewable energy offers a promising solution for mitigating climate change and addressing energy crises by converting CO_2_ into high‐value chemicals such as formic acid (HCOOH).^[^ ^1^ ^]^ HCOOH has attracted significant attention due to its high energy density and hydrogen storage potential.^[^ ^2^, ^3^ ^]^ Bismuth‐based materials demonstrate high selectivity for HCOOH due to their unique 6*p* orbital electronic structure, which favors stabilization of the *OCHO intermediate while hindering the *COOH pathway.^[^ ^4^, ^5^ ^]^ However, achieving high activity and selectivity under industrially relevant current densities remains a challenge, particularly because of the non‐ideal electronic structure and active site distribution in Bi‐based catalysts, which can promote undesired side reactions such as hydrogen evolution (HER).^[^ ^6^, ^7^, ^8^ ^]^

The catalyst microenvironment plays a crucial role in reaction selectivity by modulating the electronic density of active sites and their interactions with reaction intermediates.^[^ ^9^ ^]^ Studies have shown that coordination engineering or the introduction of functional groups can precisely adjust the microenvironment of the metal center, thereby optimizing adsorption behavior and suppressing competing reactions.^[^ ^10^, ^11^ ^]^ Single‐site catalysts (SSCs) anchor atomically dispersed active sites within ligands, providing a new strategy to address the uneven distribution of electronic density and aggregation of active sites typically observed in conventional catalysts. For example, in Bi SSCs, the coordination microenvironment directly determines the selectivity between the *OCHO and *COOH reaction pathways. Wang et al. demonstrated that the Bi‐N_2_O_2_ coordination structure facilitates electron transfer, lowers the adsorption energy of *OCHO, and enhances HCOOH production, while Wu et al. found that Bi‐NS coordination stabilizes the *COOH intermediate, leading to preferential CO production.^[^ ^12^, ^13^ ^]^ These pronounced differences highlight the necessity for precise coordination microenvironment control in HCOOH‐oriented CO_2_RR systems. Although certain Bi SSCs have been verified to effectively catalyze HCOOH production, the development of catalyst systems that simultaneously achieve facile synthesis, structural stability, and high selectivity (particularly HER suppression) remains a pivotal research challenge.^[^ ^12^, ^14^, ^15^, ^16^ ^]^

Given the significant role of coordination microenvironments, ligand engineering through the design of coordination structures has been confirmed as a crucial approach to modulating the electronic properties of the metal center. For instance, Duan et al. introduced the electronegative ‐F group into Cu SSCs, which altered the electronic state of Cu^δ+^ and shifted the CO_2_RR pathway from CO production to CH_4_ production.^[^ ^17^ ^]^ This finding suggests that rational design of the coordination microenvironment can direct the modulation of metal electronic properties, providing new insights for optimizing the selectivity of Bi SSCs.^[^ ^18^ ^]^ However, the mechanisms by which the ligand microenvironment affects the electronic structure of Bi sites and its relationship with reaction pathway selectivity remain unclear.^[^ ^19^, ^20^ ^]^ Specifically, enhancing *OCHO adsorption while suppressing the competition between the *COOH intermediate and HER remains a critical challenge for achieving high HCOOH selectivity (>95%) with low HER activity (<5%).

**Figure 1:**
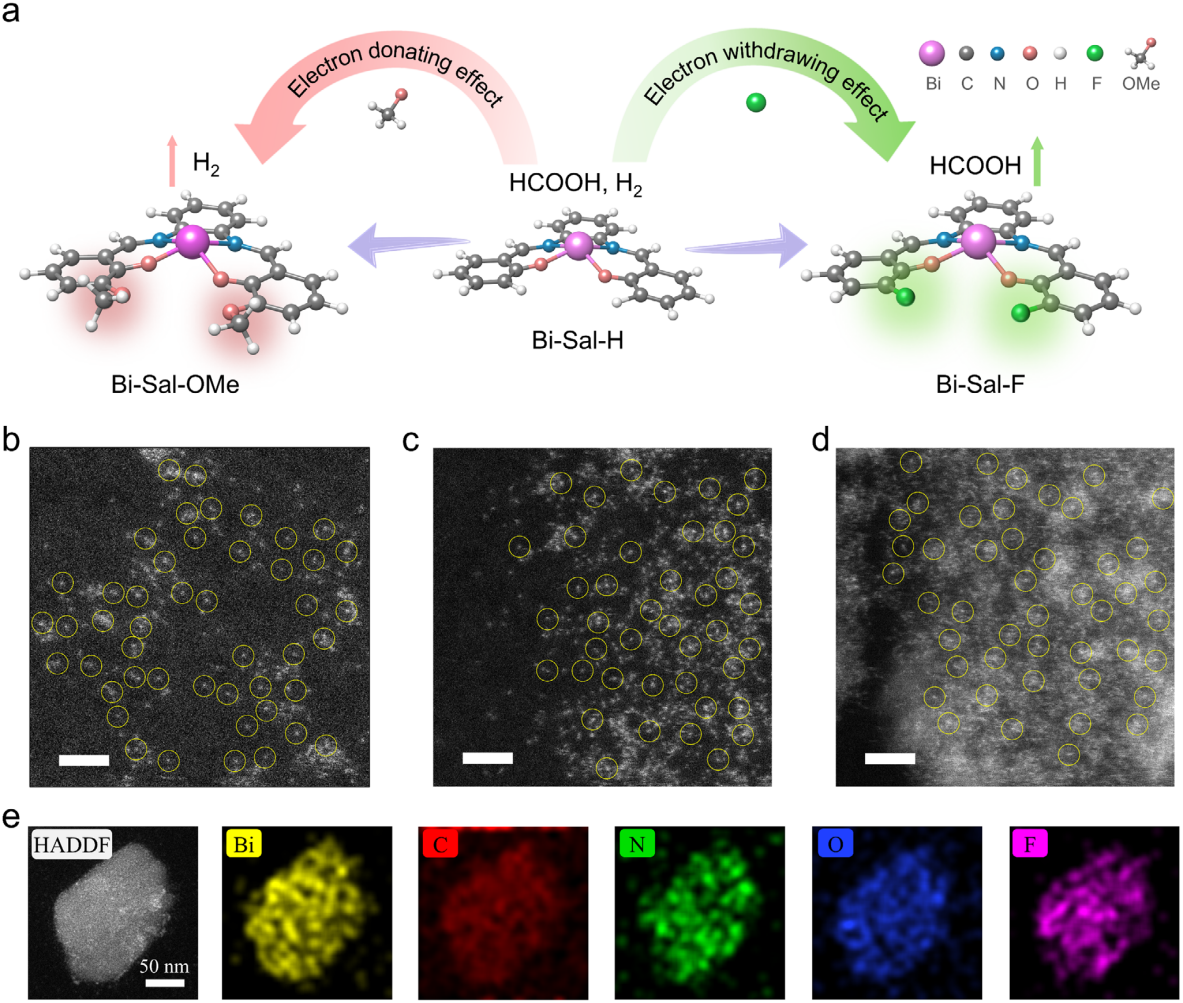
a) Mechanism of ligand microenvironment regulation in CO_2_RR over Bi‐Sal‐R catalysts. b–d) HAADF‐STEM images of Bi‐Sal‐F (b), Bi‐Sal‐H (c), and Bi‐Sal‐OMe (d), with the scale bar corresponding to 2 nm. e) Elemental mapping of Bi‐Sal‐F, showing the spatial distribution of Bi and other elements.

In this study, we modulated the microenvironment of Bi‐based SSCs by introducing various electronegative groups (R = ‐F, ‐H, and ‐OMe (methoxy group, ‐OCH_3_)) into the salophen ligand and examined their effects on CO_2_RR (**Figure** **1**a). The synthesized Bi‐Sal‐R series achieved tunable HCOOH selectivity across a wide current density range. Experimental and DFT revealed that the electron‐withdrawing ‐F group reduced the band gap and electron density of Bi, promoting oxygen‐coordinated CO_2_ adsorption. This configuration significantly lowered energy barriers for *OCHO intermediates while raising barriers for *COOH and HER. In contrast, the electron‐donating ‐OMe group exhibited weaker HER suppression, limiting HCOOH production, whereas the neutral ‐H group showed moderate HCOOH selectivity with residual H_2_ generation. Under industrial current densities (−0.1 to −0.5 A cm^−2^), Bi‐Sal‐F demonstrated exceptional performance with a Faradaic efficiency (FE) of 95% for HCOOH, while suppressing HER to an FE below 5%, and maintained stability over 17 h. Comparatively, Bi‐Sal‐H and Bi‐Sal‐OMe showed higher HER values (14% and 38%, respectively) at high current densities. These findings highlight the profound impact of SSCs microenvironment regulation on the electronic structure and catalytic performance of Bi‐based catalysts, providing crucial insights and practical guidance for the design of highly efficient CO_2_RR catalysts in the future.

## 2 Results and Discussion

### 2.1 Synthesis and characterizations

The Bi‐Sal‐R catalysts (R = ‐F, ‐H, ‐OMe) were synthesized through a coordination assembly reaction, as illustrated in Figure S1 (Supporting information).^[^ ^17^, ^21^ ^]^ In this process, salophen ligands (Sal‐R), modified with substituents of different electronegativities, form coordination bonds with Bi^3+^ ions via N_2_O_2_ chelating sites (the detailed synthesis steps in the Supporting Information). The ‐F group serves as a strong electron‐withdrawing group, the ‐OMe group as an electron‐donating group, while the ‐H group is electronically neutral. Scanning electron microscopy (SEM) images showed distinct morphologies for the catalysts, with Bi‐Sal‐F and Bi‐Sal‐H exhibited rod‐like structures, while Bi‐Sal‐OMe displays a dispersed block‐like form (Figure S2, Supporting information). The aberration‐corrected HAADF‐STEM images (Figure 1b–d and Figures S3–S5, Supporting information) show that Bi is distributed as single sites, with no Bi nanoparticles, which agrees with the X‐ray diffraction (XRD) results (Figure S7b, Supporting information). Energy‐dispersive X‐ray spectroscopy (EDS) mapping analysis shows that Bi, C, O, N, and F are homogeneously dispersed throughout the entire architecture of Bi‐Sal‐R (Figure 1e; Figure S6, Supporting information).

The molecular structures of Sal‐R and Bi‐Sal‐R were confirmed using various characterization techniques. The C═N stretching vibration band at 1620 cm^−1^ confirmed the successful condensation of the Sal‐R ligands in the FTIR spectrum (Figure S8, Supporting information).^[^ ^17^, ^22^ ^]^ The introduction of Bi^3+^ significantly enhances the UV absorption intensity without altering the peak profile of Sal‐R, suggesting that Bi may coordinate with Sal‐R (Figure S9, Supporting information).^[^ ^23^ ^]^ The coordination of the Bi^3+^ ion with the N_2_O_2_ sites in Bi‐Sal‐R was confirmed by ^1^H NMR spectroscopy (Figures S10 and S11, Supporting information). The Bi^3+^ ion content was found to be in good agreement with the theoretical values by ICP‐MS analysis (Table S3, Supporting information). Finally, the characteristic peaks for Bi‐Sal‐F, Bi‐Sal‐H, and Bi‐Sal‐OMe at m/z 559.34, 522.89, and 582.93, respectively, were in good agreement with the theoretical molecular weights by matrix‐assisted laser desorption/ionization time‐of‐flight mass spectrometry (MALDI‐TOF MS) (Figure S12, Supporting information). These results provide strong evidence for the structural accuracy of the Bi‐Sal‐R catalyst.

**Figure 2:**
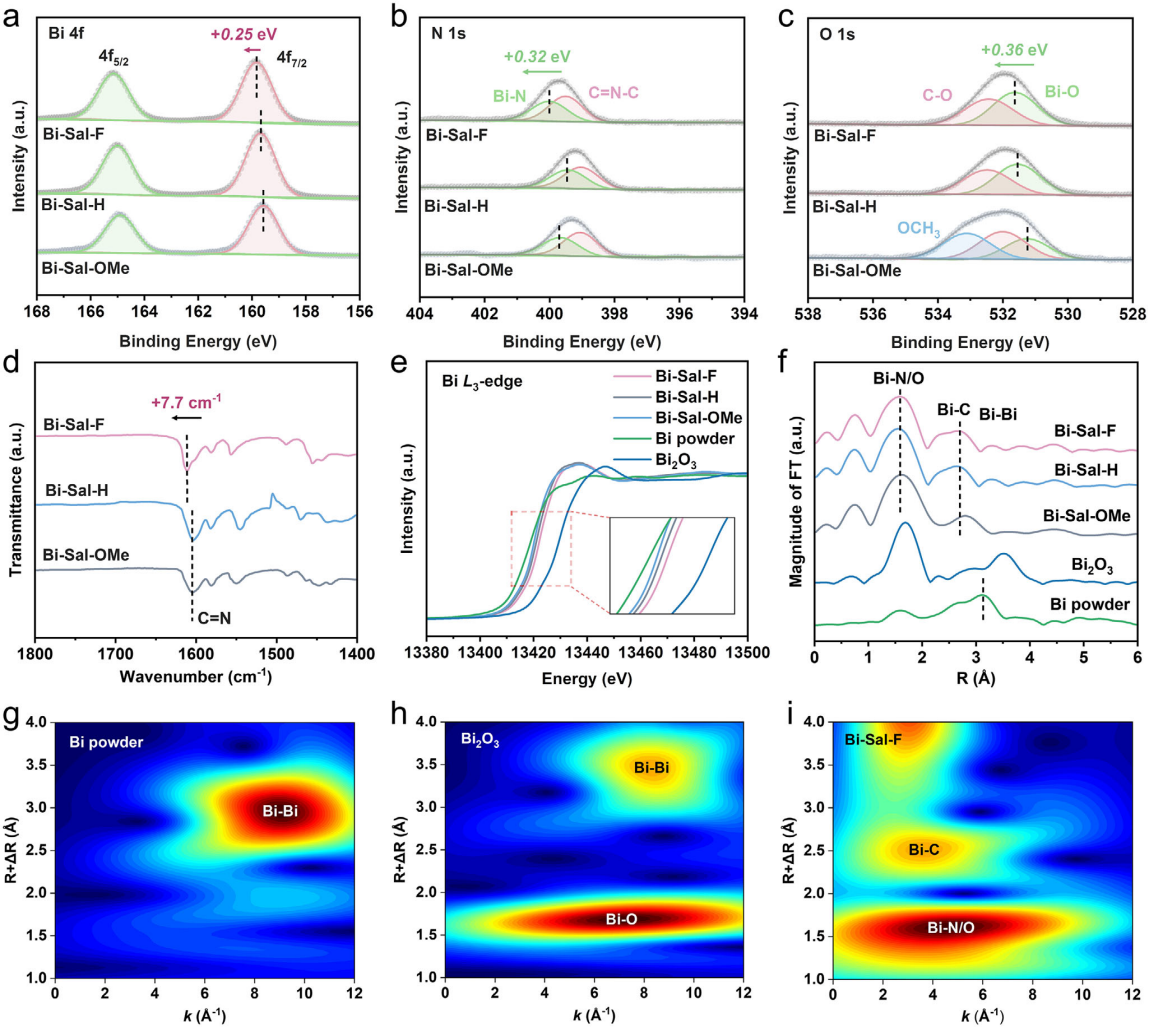
a–c) Bi 4f, N 1s and O 1s XPS spectra of Bi‐Sal‐R samples. (d) FTIR spectra of Bi‐Sal‐R samples. e) XAFS spectra at the Bi *L*_3_‐edge. f) FT‐EXAFS spectra at the Bi *L*_3_‐edge. The data are uncorrected for phase. g–i) Wavelet transform of the k^3^‐weighted EXAFS for the Bi *L*_3_‐edge in Bi powder (g), Bi_2_O_3_ (h), and Bi‐Sal‐F (i).

X‐ray photoelectron spectroscopy (XPS) was performed to analyze the impact of functional groups on the binding energy of Bi‐Sal‐R (Figure S13, Supporting information). The high‐resolution Bi 4f XPS spectrum shows peaks at ≈159.8 and ≈165.1 eV, which correspond to Bi^3+^ 4f_7/2_ and 4f_5/2_, respectively (**Figure** **2**a; Table S4, Supporting information). Under the influence of the ‐F group, the Bi 4f XPS peaks show higher binding energies (≈0.25 eV), indicating that the electron‐withdrawing effect of ‐F reduces the electron density at the Bi center, increasing its oxidation state.^[^ ^10^, ^24^ ^]^ The N 1s peak of Bi‐Sal‐R can be deconvoluted into Bi‐N (400.0 eV) and C═N─C (399.5 eV), with the N 1s peak in Bi‐Sal‐F exhibiting a 0.32 eV shift toward higher binding energies (Figure 2b).^[^ ^12^ ^]^ Similarly, the O 1s peak can be deconvoluted into C─O (532.4 eV) and Bi─O (531.6 eV), with a 0.36 eV shift observed for the Bi─O binding energy in Bi‐Sal‐F (Figure 2c).^[^ ^12^ ^]^ FTIR analysis further confirmed the shift of ‐N═C‐ bond absorption peak to higher wavenumbers (Figure 2d), revealing that the functional group substitution alters the electronic environment and therefore reducing the electron‐donating ability of ligand toward the central Bi site. The results indicate that the ‐F group not only influences the oxidation state of Bi but also alters the charge distribution across the entire molecule, indicating the significant role of the ‐F group in regulating both the Bi center.

X‐ray absorption spectroscopy (XAS) was used to further analyze the electronic structure and coordination environment of Bi‐Sal‐R. Figure 2e shows the X‐ray absorption near‐edge structure (XANES) spectrum of Bi *L*_3_‐edge. The near‐edge absorption energy of Bi‐Sal‐R falls between those of Bi powder and Bi_2_O_3_, indicating that the oxidation state of Bi in Bi‐Sal‐R is between Bi^0^ and Bi^3+^.^[^ ^12^ ^]^ Compared to Bi‐Sal‐H and Bi‐Sal‐OMe, the near‐edge absorption energy of Bi‐Sal‐F shifts slightly to higher energy, suggesting a higher oxidation state due to the electron‐withdrawing effect of the ‐F group. The Fourier transform extended X‐ray absorption fine structure (EXAFS) spectra of Bi‐Sal‐R (Figure 2f) exhibit two distinct peaks corresponding to the Bi‐N/O and Bi‐C scattering paths, attributed to the Bi‐N_2_O_2_ coordination structure and the distal aromatic C, respectively. The fitting results for Bi‐Sal‐R indicate a coordination number of ≈4.0 for the Bi‐N/O path and ≈6.3 for the Bi‐C path, with no Bi‐Bi coordination detected.^[^ ^6^, ^17^ ^]^ The average lengths of the Bi─N/O and Bi─C bonds are estimated to be 2.21 and 3.21 Å, respectively, with detailed fitting parameters provided in Figure S14 and Table S5 (Supporting information). Further wavelet transform EXAFS analysis of the k‐space data confirms the absence of Bi‐Bi coordination and reveals differences in the Bi‐O coordination (Figure 2g–i; Figure S15, Supporting information). This result is consistent with the expected molecular coordination environment, confirming the successful synthesis of the designed structures.

### 2.2 CO_2_RR Performance

To clarify the correlation between electronic structure and product selectivity, we assessed the CO_2_RR performance using a three‐compartment flow cell reactor (Figure S16, Supporting information). Gaseous and liquid products were quantified by gas chromatography and liquid chromatography, respectively, with calibration curves established using standard concentration gases and standard HCOOH solutions (Figures S17 and S18, Supporting information). Linear sweep voltammetry (LSV) was employed to analyze the catalytic response under CO_2_ and Ar conditions (Figure S19, Supporting information). The larger current density difference between CO_2_ and Ar conditions underscores the superior CO_2_RR kinetics of Bi‐Sal‐F. Bi‐Sal‐F exhibited a more positive onset potential compared to the other samples, highlighting its rapid initiation of CO_2_RR.

**Figure 3:**
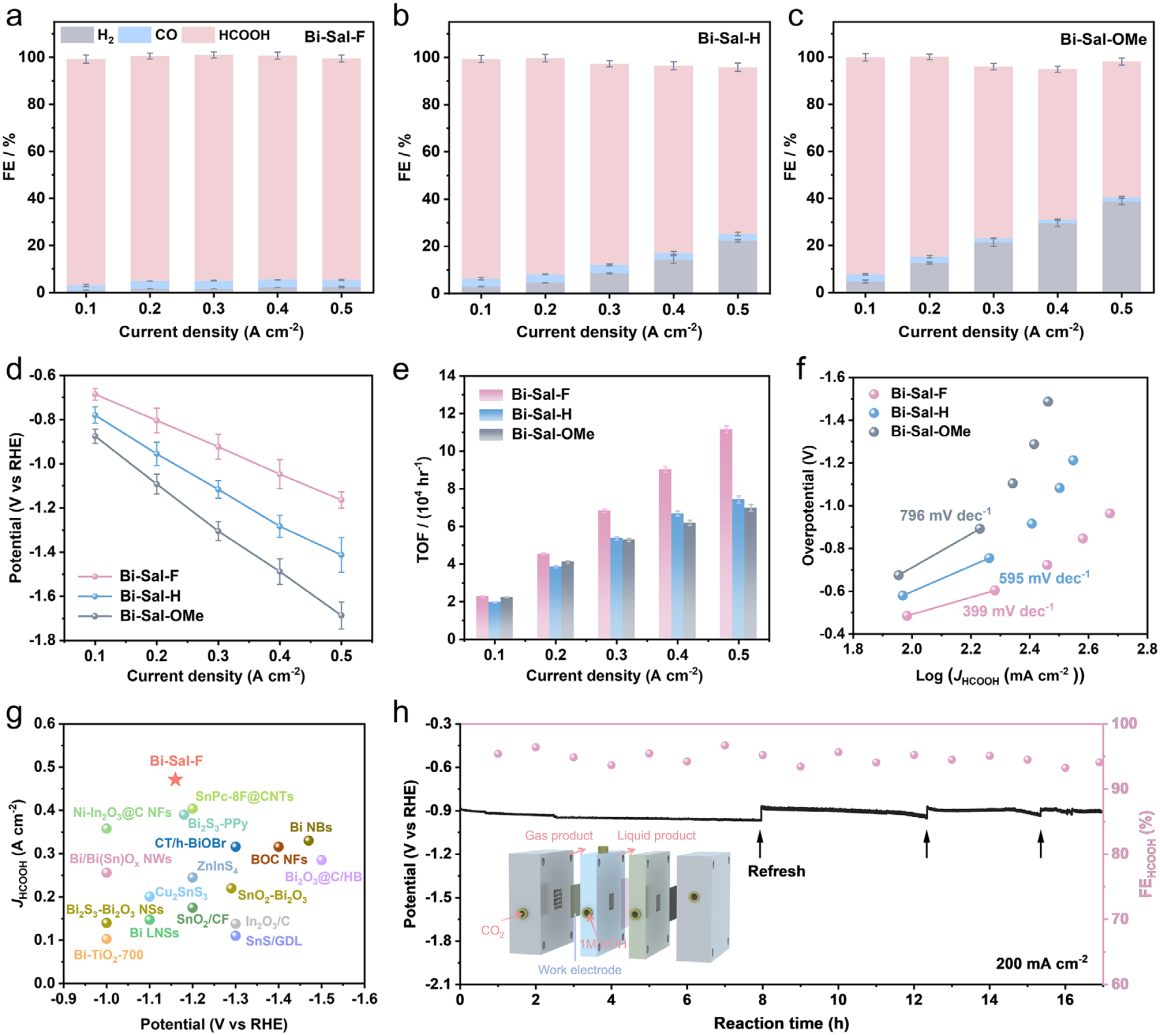
Electrochemical CO_2_RR performance. a–c) FE for all products on (a) Bi‐Sal‐F, (b) Bi‐Sal‐H, and (c) Bi‐Sal‐OMe in 1 M KOH electrolyte. d) Voltage (V vs RHE) as a function of current density. e) TOF and f) Tafel plots for Bi‐Sal‐R. g) Comparison with the performance of the current catalysts. h) Stability test of Bi‐Sal‐F at a current density of 200 mA cm^−2^.

We next analyzed the product distribution and FE of Bi‐Sal‐R catalysts over a range of current densities, from −0.1 to −0.5 A cm^−2^. Bi‐Sal‐F, with the ‐F substituent, showed strong suppression of HER while maintaining ≈95% HCOOH selectivity across all current densities tested (**Figure** **3**a). By comparison, Bi‐Sal‐H exhibited lower H_2_ selectivity than Bi‐Sal‐F but still produced substantial amounts of H_2_ at high current densities (Figure 3b). On the other hand, Bi‐Sal‐OMe showed increasing H_2_ selectivity at higher current densities, while HCOOH selectivity dropped significantly. At −0.5 A cm^−2^, Bi‐Sal‐OMe achieved ∼58% HCOOH selectivity (Figure 3c). The exceptional performance of Bi‐Sal‐F is reflected in its low overpotential across a current density range of −0.1 to −0.5 A cm^−2^ (Figure 3d). At −0.1 A cm^−2^, it requires only −0.69 V versus RHE, while at ‐0.5 A cm^−2^, the working voltage is as low as −1.16 V versus RHE. In comparison, Bi‐Sal‐H and Bi‐Sal‐OMe require −1.41 and −1.69 V versus RHE, respectively, demonstrating that Bi‐Sal‐F significantly reduces the energy demand for catalysis. We further evaluated the performance of the three catalysts in the electrochemical reduction of CO_2_ to HCOOH. At a current density of −0.5 A cm^−2^, Bi‐Sal‐F achieved a partial current density for HCOOH production of −0.471 A cm^−2^, significantly surpassing Bi‐Sal‐H (−0.353 A cm^−2^) and Bi‐Sal‐OMe (−0.29 A cm^−2^), indicating its superior activity for HCOOH generation (Figure S20, Supporting information).

To investigate the influence of group electronegativity on the CO_2_RR activity, we analyzed kinetic parameters including turnover frequency (TOF), Tafel slope, double‐layer capacitance (C_dl_), and charge transfer resistance. The results demonstrate that Bi‐Sal‐F exhibits higher TOF values across the entire current range (Figure 3e). Furthermore, Bi‐Sal‐F shows a significantly lower Tafel slope (399 mV dec^−1^) compared to Bi‐Sal‐H (595 mV dec^−1^) and Bi‐Sal‐OMe (796 mV dec^−1^), indicating faster reaction kinetics (Figure 3f). C_dl_ measurements and Nyquist plot analysis reveal that Bi‐Sal‐F possesses a larger electrochemical active surface area and the smallest charge transfer resistance (Figure S21 and S22 and Table S6, Supporting information).^[^ ^25^ ^]^ These findings collectively suggest that Bi sites modified with electron‐withdrawing groups can significantly enhance CO_2_RR activity by improving electron transfer efficiency and optimizing reaction kinetics.

When benchmarked against other high‐performance CO_2_RR catalysts reported in the literature, Bi‐Sal‐F exhibited notable HCOOH production performance (Figure 3g; Table S7, Supporting information). Most importantly, Bi‐Sal‐F showed remarkable long‐term stability during continuous electrolysis at ‐200 mA cm^−2^, with minimal fluctuations in operating voltage and a consistently high FE_HCOOH_ exceeding 93% over 17 h of operation (Figure 3h).

The structure of the catalyst was characterized after prolonged CO_2_RR testing, with a focus on the stability of the Bi single‐site structure. XRD analysis revealed that the diffraction peaks of the post‐electrolysis electrode closely matched those of bare carbon paper, with no detectable characteristic peaks corresponding to Bi particles or Bi_2_O_2_CO_3_ (Figure S23, Supporting information), ruling out the possibility of Bi aggregation or phase transformation. FTIR spectroscopy further confirmed the molecular structural stability of the catalyst, as the characteristic peaks of the C═N bond remained unchanged after both alkaline immersion and electrolysis (Figure S24, Supporting information), indicating that the salophen molecular structure remained intact. XPS analysis demonstrated that the binding energies and peak profiles of Bi, C, N, and O elements showed no significant changes after electrolysis (Figure S25, Supporting information), confirming the stable oxidation state of Bi before and after the reaction. These results collectively demonstrate that the Bi‐Sal‐F exhibits reasonable structural stability during prolonged electrolysis.

### 2.3 Mechanistic insights of Bi‐Sal‐F

**Figure 4:**
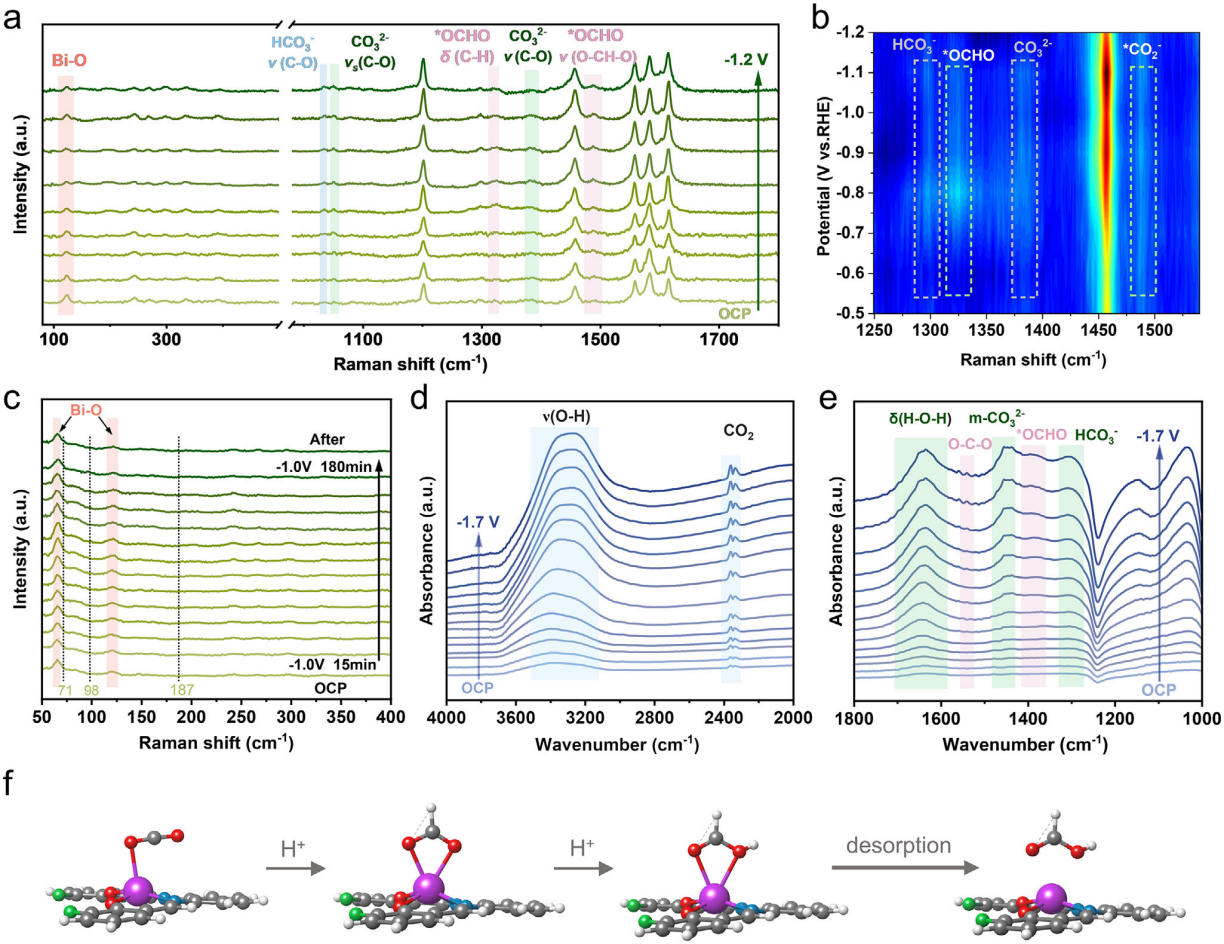
a) In situ Raman spectra of Bi‐Sal‐F, collected from OCP to −1.2 V versus RHE. b) 2D color mapping of in situ Raman spectra of Bi‐Sal‐F. c) In situ Raman spectra of the Bi‐Sal‐F catalyst recorded during 3‐h electrolysis at −1.0 V versus RHE. d,e) In situ FTIR spectra of Bi‐Sal‐F, recorded over the potential range from OCP to −1.7 V s RHE. f) Proposed reaction pathway for HCOOH production on Bi‐Sal‐F.

We employed in situ Raman spectroscopy and in situ ATR‐FTIR spectroscopy to investigate the stability of the catalyst structure and the reaction intermediates during the CO_2_RR, aiming to provide a deeper understanding of the CO_2_RR mechanism. Under open‐circuit potential (OCP), the spectrum primarily reflects the intrinsic features of the molecular structure. A peak at 1202 cm^−1^ corresponds to both the C‐H bending (δ C─H) and C═C stretching (ν C═C) vibrations of the benzene ring (**Figure** **4**a).^[^ ^26^ ^]^ Additional peaks at 1457, 1558, and 1584 cm^−1^ are also linked to the benzene ring, representing channel relaxation and further C═C stretching modes.^[^ ^27^, ^28^ ^]^ The peak at 1614 cm^−1^ is attributed to the characteristic vibration of C═N bonds.^[^ ^27^ ^]^ Importantly, these spectral features remained essentially unchanged throughout the CO_2_RR process, suggesting that the molecular structure is well preserved. In particular, the stability of the C═N bond indicates that the salophen ligand remains intact during electrolysis, thereby providing a stable platform for modulating the metal center's catalytic activity. To further assess the structural integrity of the Bi single sites under operating conditions, long‐term electrolysis coupled with in situ Raman monitoring was performed (Figure 4c). Characteristic signals for Bi‐O coordination at 62 and 119 cm^−1^ persisted during electrolysis at −1.0 V versus RHE, while no peaks corresponding to metallic Bi (E_g_ and A_1g_ modes at 71 and 98 cm^−1^) or Bi_2_O_2_CO_3_ (at 187 cm^−1^) were detected.^[^ ^29^, ^30^ ^]^ These results confirm the high structural stability of Bi‐Sal‐F during electrochemical CO_2_RR.

To elucidate the microscopic mechanism of the catalytic reaction pathway, the adsorption states of intermediate species on the catalyst surface were systematically analyzed (Figure 4a,b). The characteristic peaks observed at 1033 cm^−1^, 1051 cm^−1^, and 1383 cm^−1^ can be unequivocally assigned to the C─O vibrational modes of chemically adsorbed HCO_3_^−^ and CO_3_^2−^ species.^[^ ^30^, ^31^, ^32^ ^]^ Notably, the C─H deformation vibration peak at 1320 cm^−1^ and the O‐(CH)‐O stretching vibration peak at 1489 cm^−1^ provide direct evidence for the presence of the *OCHO intermediate.^[^ ^33^, ^34^ ^]^ Under applied negative potential, the intensity of the C─H vibration of *OCHO significantly weakens, indicating its consumption during the reduction reaction, which originates from the *OCHO‐to‐HCOOH transformation driven by proton‐coupled electron transfer. As the voltage increases, more molecules can overcome the activation energy barrier of this reaction.^[^ ^31^ ^]^ Furthermore, the absence of characteristic peaks for the *COOH intermediate (typically at ∼1130 cm^−1^) suggests that the catalytic system significantly suppresses the CO pathway, consistent with the low CO selectivity observed in electrocatalytic tests, further confirming the excellent selectivity of the catalyst toward the HCOOH pathway.^[^ ^33^, ^35^ ^]^

We further investigated the catalytic intermediates and reaction pathways using in situ ATR‐FTIR spectroscopy (Figure S27, Supporting information). As shown in Figure 4d‐e, the bands at 1540 and 1380 cm^−1^ correspond to the O─C─O asymmetric stretching vibration and *OCHO species, respectively.^[^ ^36^ ^]^ A weak HCOOH signal is detected at 1560 cm^−1^.^[^ ^12^ ^]^ This indicates that the Bi sites adsorb CO_2_ via two oxygen coordination rather than one carbon coordination, which is consistent with the *OCHO‐to‐HCOOH pathway, rather than the *COOH‐to‐CO pathway.^[^ ^33^ ^]^ Additionally, no typical *CO signals (1900‐2100 cm^−1^) were observed during the electrocatalytic process, indicating that CO formation on the catalyst surface is negligible and HCOOH is the predominant product.^[^ ^5^ ^]^ The peak at 1448 cm^−1^ corresponds to monodentate carbonate (m‐CO_3_^2−^) groups. The peak at 1650 cm^−1^ corresponds to the adsorption of H_2_O.^[^ ^10^ ^]^ As the voltage becomes more negative, the increase in the H_2_O signal suggests that Bi may facilitate the dissociation of H_2_O, which is likely involved in the hydrogenation of CO_2_.^[^ ^37^ ^]^

In situ Raman and ATR‐FTIR analysis elucidated the reaction pathway and confirmed the structural stability of Bi‐Sal‐F during CO_2_RR. Bi‐Sal‐F maintained its SSCs structure throughout the catalysis. We observed the presence of the *OCHO intermediate, while the *COOH intermediate and *CO product were scarcely detected. This result further explains the selective formation of HCOOH by Bi‐Sal‐F, rather than CO. Based on these findings, we propose a reaction mechanism in which the Bi site first adsorbs CO_2_ through oxygen‐coordination. Subsequently, H_2_O dissociate at the Bi site to provide *H, facilitating the formation of the *OCHO intermediate. This intermediate is then converted into *HCOOH, which is subsequently released to complete the catalytic cycle (Figure 4f).

### 2.4 DFT calculations

**Figure 5:**
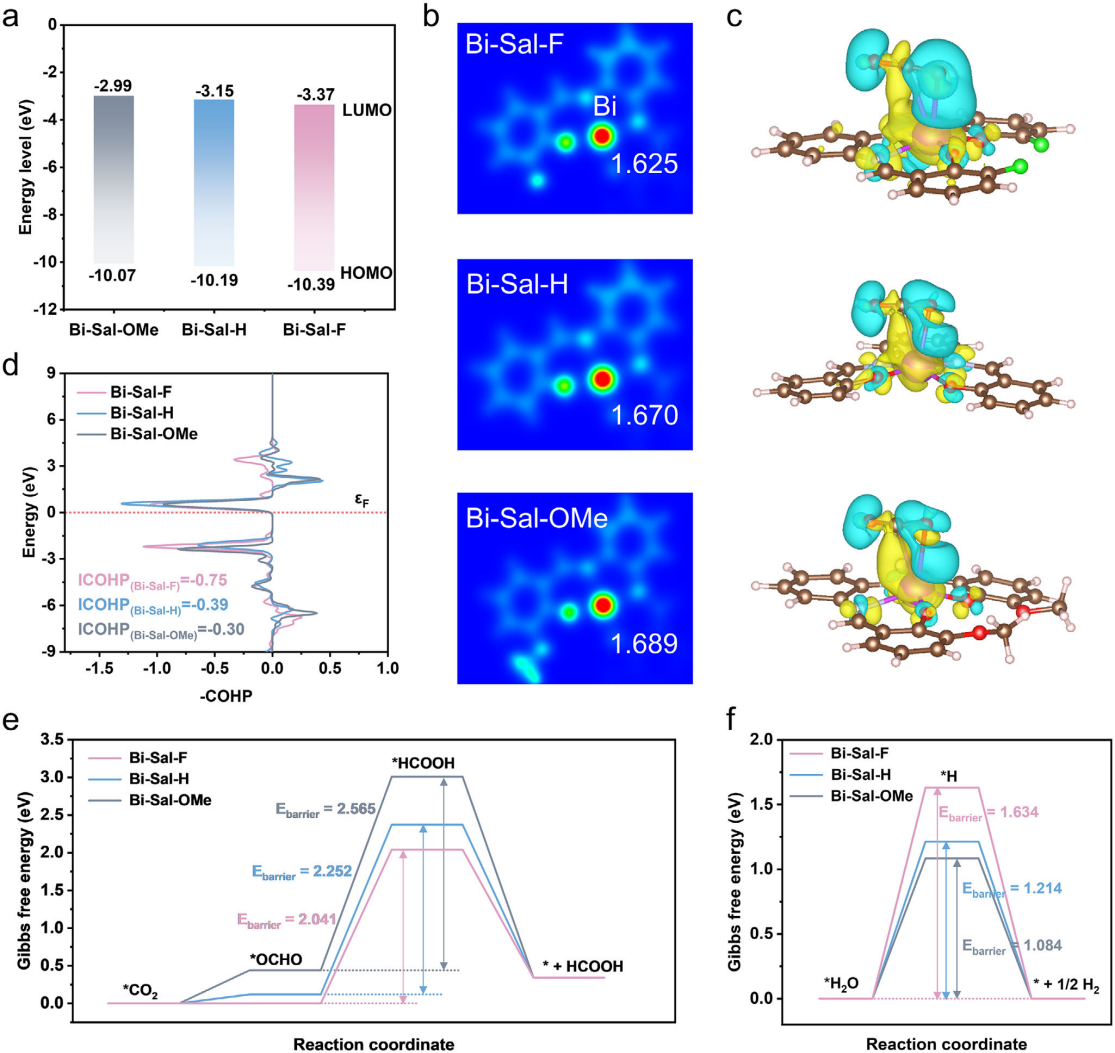
a) Calculated energy levels of the HOMO and LUMO for Bi‐Sal‐R. b) Charge density maps for Bi‐Sal‐R, with cyan and orange regions indicating electron accumulation and depletion, respectively. c) Differential charge density of CO_2_ adsorption on Bi‐Sal‐R catalysts, where yellow and blue regions represent electron accumulation and depletion, respectively. d) ‐COHP curves for CO_2_ adsorption on Bi‐Sal‐R catalysts. Negative values (‐COHP<0) correspond to bonding interactions, while positive values (‐COHP>0) indicate anti‐bonding interactions. The Fermi level (E_F_) is set at 0 eV. e,f) Free energy diagrams for the CO_2_RR and HER on Bi‐Sal‐R, illustrating the production of e) HCOOH and f) H_2_.

To investigate the regulatory effect of ligand microenvironments on the electronic structure of Bi‐Sal‐R catalysts, we constructed three models modified with ‐F, ‐H, and ‐OMe groups (Figure S28a, Supporting information). Analysis of the LUMO and HOMO energy levels revealed that the introduction of the electron‐withdrawing ‐F group lowered the energy levels of Bi‐Sal‐F to −3.37 and −10.39 eV, respectively, resulting in a narrower band gap that facilitates electron excitation (**Figure** **5**a). Meanwhile, the electron density at the Bi center decreased to +1.625 eA^−3^, enhancing its electrophilicity (Figure 5b), which is favorable for accepting electrons and transferring them to CO_2_. In contrast, electron‐donating ‐OMe group induced the opposite effect, raising the LUMO and HOMO levels to −2.99 and ‐10.07 eV, and increasing the Bi site electron density to + 1.689 eA^−3^. This may weaken the electrophilicity of Bi and reduce its ability to activate CO_2_. These electronic structure variations are consistent with the observed trends in catalytic performance.

We investigated the electronic interactions between the Bi‐Sal‐F catalyst and adsorbed CO_2_ to better understand its CO_2_ adsorption and activation ability. The differential charge density map reveals significant electron depletion (blue regions) around the oxygen atoms of CO_2_ adsorbed on Bi‐Sal‐F, indicating electron transfer from Bi to CO_2_ (Figure 5c). Crystal Orbital Hamilton population (‐COHP) analysis shows that Bi‐Sal‐F exhibits stronger bonding interactions with CO_2_ in the negative energy range (Figure 5d). This trend is also reflected in the integrated COHP (ICOHP) values, where Bi‐Sal‐F (−0.75) is significantly lower than Bi‐Sal‐H (−0.39) and Bi‐Sal‐OMe (−0.30), indicating a stronger interaction with CO_2_.^[^ ^17^, ^38^ ^]^ Additionally, the PDOS analysis (Figure S30, Supporting information) shows that the occupied states of Bi‐6p orbitals in Bi‐Sal‐F (−0.95 eV) are closer to the Fermi level compared to Bi‐Sal‐H (‐1.51 eV) and Bi‐Sal‐OMe (−1.60 eV), reflecting more active electronic states.^[^ ^39^, ^40^ ^]^ Furthermore, the upward shift of the O‐p orbitals of adsorbed CO_2_ in Bi‐Sal‐F relative to Bi‐Sal‐OMe further indicates that the ‐F group enhances CO_2_ adsorption.^[^ ^17^ ^]^ In conclusion, Bi‐Sal‐F significantly promotes CO_2_ adsorption and electron sharing through enhanced electronic coupling, providing favorable conditions for the subsequent reaction.

To evaluate the catalytic performance of Bi‐Sal‐R catalysts, we calculated the free energies of reaction intermediates (Figure 5e; Table S8, Supporting information). The reaction pathway was established based on in situ experimental observations, involving the adsorption of CO_2_, formation of *OCHO and *HCOOH intermediates, and final desorption of HCOOH. Bi‐Sal‐F exhibits the lowest free energy barrier for *OCHO formation, consistent with its shortest Bi─O bond (2.50 Å) and largest CO_2_ bending angle (174.5°), both of which favor CO_2_ adsorption and activation (Figure S28d–f; Table S9, Supporting information).^[^ ^12^ ^]^ Furthermore, Bi‐Sal‐F achieves the lowest free energy barrier (2.041 eV) in the rate‐limiting step (*OCHO to *HCOOH), making it significantly more efficient than Bi‐Sal‐H and Bi‐Sal‐OMe. During the *HCOOH desorption step, Bi‐Sal‐F shows a smoother energy profile, indicating its superior catalytic performance. This can be attributed to the longer adsorption distance between Bi and *HCOOH, which facilitates easier desorption (Figure S29d–f; Table S10, Supporting information).^[^ ^41^ ^]^

Finally, we evaluated the free energy changes for the competing HER and CO pathways. Bi‐Sal‐F exhibits the highest energy barrier (1.634 eV) for *H formation, indicating lower HER activity (Figure 5f; Table S11, Supporting information). Further analysis of the Δ*U*_L_ (*U*_L_(CO_2_RR) − *U*_L_(HER)) reveals that Bi‐Sal‐F has the smallest Δ*U*_L_ value, suggesting a thermodynamic preference for CO_2_RR leading to HCOOH formation rather than HER (Figure S31 and Table S12, Supporting information).^[^ ^12^ ^]^ Additionally, Bi‐Sal‐F shows a higher free energy barrier along the *COOH pathway, indicating a stronger tendency to form the *OCHO intermediate (Figure S32 and Table S13, Supporting information). These results suggest that, despite Bi‐Sal‐F being an SSC structure, its low Faradaic efficiency for CO production is mainly due to its preferential selectivity toward the HCOOH formation pathway.

DFT calculations demonstrate that substituent electronegativity effectively tunes the electronic structure and catalytic selectivity of Bi SSCs. Specifically, the electron‐withdrawing ‐F group lowers the energy levels of Bi‐Sal‐F, reduces the electron density at the Bi center, and enhances its bonding interactions with CO_2_, thereby promoting CO_2_ adsorption and activation. In contrast, the ‐H and ‐OMe groups have a milder effect on the Bi electronic structure, which is less favorable for CO_2_ adsorption and activation. Furthermore, the calculated free energies along the reaction pathways reveal that Bi‐Sal‐F not only facilitates water splitting to generate *H but also suppresses HER, while its lower energy barrier along the *OCHO pathway (relative to the *COOH pathway) thermodynamically rationalizes the high selectivity for HCOOH formation.

## 3 Conclusion

We constructed a Bi‐Sal‐R (R = ‐F, ‐H, ‐OMe) catalyst system with tailored electronic microenvironments through ligand engineering. Material characterization confirms the modulation of Bi oxidation state by the electronegative group. Under industrial current densities (−0.1 to −0.5 A cm^−2^), Bi‐Sal‐F achieves 95% FE for HCOOH with stable operation over 17 h, whereas Bi‐Sal‐H and Bi‐Sal‐OMe exhibit significantly increased FE for HER (14% and 38%, respectively). DFT calculations reveal that the electron‐withdrawing effect of the ‐F group reduces the band gap and electron density of Bi, while modulating the bond length and bond angle between Bi and CO_2_ molecules. This significantly enhances CO_2_ adsorption and electron transfer at the Bi center. Additionally, the modified Bi sites promote CO_2_RR while greatly suppressing both the *COOH pathway and HER. The reaction pathway analysis shows that Bi‐Sal‐F exhibits highly favorable selectivity for HCOOH with a low energy barrier, highlighting the crucial role of electronic tuning in improving catalyst performance. This study not only underscores the importance of electronic tuning of microenvironment in enhancing the efficiency and selectivity of CO_2_RR but also provides a versatile platform for exploring structure‐activity relationships of other electrochemical applications by modulating the microenvironment between the metal center and ligand.

## Conflict of Interest

The authors declare no conflict of interest.

## Supporting information



Supporting Information

## Data Availability

Research data are not shared.
