# Supplementary material for: Electronic Microenvironment Regulation of Bismuth‐salophen Single‐site Catalysts for Enhanced Selectivity in CO2 Electrolysis to Formic Acid
Source: Adv Sci (Weinh). 2025 Jun 10;12(31):e02061. doi: 10.1002/advs.202502061 (PMC12376708; doi:10.1002/advs.202502061)
Supplement: Supplementary file 1 — Supporting Information [file ADVS-12-e02061-s001.docx]

**Supporting Information**

**Electronic Microenvironment Regulation of Bismuth-salophen Single-site Catalysts for Enhanced Selectivity in CO_2_ Electrolysis to Formic Acid**

Tianxing Wang^1, a, b, c^, Tian (Leo) Jin^1, b, d, *^, Zhiping Liu^b^, Jingtao Wang^b^, Yue Gong^e^, Ming Ma^f^, Jie Chen^f, *^, Shaohua Shen^g^, Rongqian Wu^a, b, c^, Yu-Cheng Huang^h^, Ying Rui Lu^h^, Yi Lyu^a, b, c, *^, Xiaofei Liu^a, b, c, *^

^a^ Department of Hepatobiliary Surgery, First Affiliated Hospital of Xi’an Jiaotong University, Xi’an 710049, China

^b^ Center for Regenerative and Reconstructive Medicine, Med-X Institute, the First Affiliated Hospital of Xi’an Jiaotong University, Xi’an 710049, China

^c^ School of Future Technology, Xi'an Jiaotong University, Xi’an 710049, China

^d^ Department of Applied Chemistry, School of Chemistry, MOE Key Laboratory for Nonequilibrium Synthesis and Modulation of Condensed Matter, Xi’an Key Laboratory of Sustainable Energy Materials Chemistry, State Key Laboratory of Electrical Insulation and Power Equipment, State Key Laboratory for Mechanical Behavior of Materials, Xi’an Jiaotong University, Xi’an 710049, China

^e^ CAS Key Laboratory of Standardization and Measurement for Nanotechnology, CAS Center for Excellence in Nanoscience, National Center for Nanoscience and Technology, Beijing 100190, China

^f^ School of Chemical Engineering and Technology, Xi’an Jiaotong University, Xi’an 710049, China

^g^ International Research Center for Renewable Energy, State Key Laboratory of Multiphase Flow in Power Engineering, Xi’an Jiaotong University, Xi’an 710049, China

^h^ National Synchrotron Radiation Research Center, Hsinchu 300092, Taiwan

^1^ These authors contributed equally to this work.

^*^ Correspondence: xiaofeiliu@xjtu.edu.cn (X.L.), tianjin.ecust@gmail.com (T. L. Jin), jie.chen@xjtu.edu.cn (J. C.)

**1. Experimental section**

**Materials.:** Bi(NO_3_)_3_⋅5H_2_O (99%), 1,2-phenylenediamine (99%), salicylaldehyde (99%), 3-fluoro-2-hydroxybenzaldehyde (99%), 3-methoxysalicylaldehyde (99%), and potassium hydroxide (KOH, 85%) were obtained from Aladdin Co., Ltd. Methanol (MeOH, AR) and isopropanol (AR) were procured from Tianjin Fuyu Chemical Co., Ltd. Nafion 117 solution (5 wt.%) was supplied by Admas Co., Ltd. Ultrapure water was obtained from the home-made purification system. All chemicals were used without further purification.

**Synthesis of Sal-R complexes:** First, 3 mmol of 1,2-phenylenediamine and 6 mmol of 3-Fluoro-2-hydroxybenzaldehyde were individually added into 15 mL of methanol. After ultrasonic dissolution, the solutions were thoroughly mixed and refluxed under at 60 ºC for 4 hours. After allowing the solution to cool down to room temperature, the precipitate was filtered, washed with methanol, and dried at 60 ºC to obtain the Sal-F complex. Similarly, the Sal-H complex and the Sal-OMe complex were synthesized through the substitution of 3-Fluoro-2-hydroxybenzaldehyde with Salicylaldehyde and 3-Methoxysalicylaldehyde, respectively.

**Synthesis of Bi-Sal-R catalysts:** 0.8 mmol of Sal-F were dissolved in 20 ml of methanol through stirring, and 0.75 mmol of Bi(NO_3_)_3_⋅5H_2_O was dissolved in 10 ml of methanol. Subsequently, the methanol solution of Bi(NO_3_)_3_⋅5H_2_O was slowly added dropwise to the methanol solution of Sal-F, and the mixture was stirred at 60 ºC for 2 hours. Finally, the products were separately filtered, washed with methanol, and then dried to obtain the Bi-Sal-F. Similarly, the Bi-Sal-H catalyst and Bi-Sal-OMe catalyst were synthesized through the substitution of Sal-F with Sal-H and Sal-OMe, respectively.

**2. Characterizations**

The powder X-ray diffraction (XRD) patterns of the catalysts were recorded on a Shimadzu XRD-6100 diffractometer using Cu Kα radiation (λ=1.54 Å, 30 kV). Fourier transform infrared (FTIR) spectra were measured on a Thermo Fisher Nicolet iS50 spectrometer. The morphology of the electrocatalysts was analyzed using a field emission scanning electron microscope (FE-SEM, Hitachi S-4800) at 15 kV. The HAADF-STEM images were acquired in STEM mode using a Cs-corrected TEM (FEI Themis Z) operated at 200 kV. Energy dispersive X-ray spectrometry (EDS) was simultaneously performed to analyze the elemental distribution. X-ray photoelectron spectroscopy (XPS) data were collected using a Thermo Fisher Scientific ESCALAB Xi+ instrument. Inductively coupled plasma mass spectrometry (ICP-MS) analysis was conducted on a PerkinElmer NexION 350D system. Prior to Bi content determination, samples were calcined at 800°C for half an hour in a muffle furnace and extracted with aqueous nitric acid. Matrix-assisted laser desorption/ionization time-of-flight mass spectrometry (MALDITOF-MS) analysis was performed on a Bruker Autoflex III smartbeam instrument. The solid-state UV-vis spectra were measured using a Shimadzu UV-2600 spectrophotometer. Nuclear magnetic resonance (NMR) spectra were recorded on a Bruker AVANCE AV III 400 spectrometer to analyze the structures of ligands and catalysts. Gas-phase products were analyzed on a Tianmei GC7980 gas chromatograph equipped with FID and TCD detectors, using argon as the carrier gas. Liquid-phase products were analyzed on a Shimadzu LC-2050 liquid chromatograph using 0.05 % phosphoric acid and 95% acetonitrile as the mobile phase. All the electrochemical experiments were conducted on the electrochemical workstation (CHI 760e), from Chenhua Instrument Co., Ltd. and the Model 2461 workstation from Keithley Instruments.

XAS measurements and analysis: X-ray absorption spectroscopy (XAS), including X-ray absorption near-edge spectra (XANES) and extended X-ray absorption fine structure (EXAFS), was collected in fluorescence mode at the BL32A beamline of Taiwan Photon Source (TPS), National Synchrotron Radiation Research Center (NSRRC). The fly-Scanning XANES and EXAFS measurements were made at the Taiwan Photon Source (TPS), NSRRC. The Tender X-ray absorption spectroscopy beamline, TPS 32A, is a Tender X-ray bending magnet beamline that covers the energy range 1.7-10 keV. A full fly-Scanning XAFS spectrum can be obtained in less than 15s over an energy range of more than 800 eV. XAS measurements at the Bi *L*_3_-edge (13419 eV) were conducted in transmission mode, ensuring high sensitivity for examining the local environment of bismuth atoms. XANES data were processed following standard procedures, which included background subtraction, normalization of the edge jump, and correction for any systematic effects. This processing is critical to accurately determine the oxidation state and electronic structure of the target atoms. The data collected were normalized to the incoming incident photon ux and processed with the Athena software from the IFEFFIT package. In the analysis of EXAFS data, k^3^-weighting was applied during the Fourier transformation to emphasize the higher-k region oscillations. For fitting purposes, the k-range was selected from 3 to 12 Å^-1^, providing an adequate k-space range to enhance the quality of the signal and ensure the reliability of the fitting results. The R-range for the Fourier transform was set between 1.0 and 3.5 Å, covering the first and second coordination shells to effectively fit the nearest neighbor information. During the fitting process, theoretical scattering phase and amplitude functions were generated using the FEFF code, accurately describing the multiple scattering effects and the structural parameters between the central atom and its neighboring atoms. The fitting parameters included coordination number (N), bond length (R), Debye-Waller factor (σ^2^), and energy shift (ΔE_0_). The coordination number fitting was used to determine the number of neighboring atoms around each target atom, while the bond length represented the distance between the central atom and neighboring atoms. The Debye-Waller factor accounted for thermal and static disorder in the system, and the energy shift was used to correct the difference between the theoretical model and experimental data.

**3. Electrochemical measurements**

**The preparation of electrodes.** For preparing the catalyst ink of Bi-Sal-R, 7 mg of catalyst and 2.5 mg of carbon black were mixed with 1950 μL of isopropanol and 50 μL of 5 wt.% Nafion solution, followed by intensive ultrasonication to obtain the catalyst ink with a concentration of 3.5 mg mL^-1^. Then, 350 μL of catalyst ink was drop-casted onto a 1.5×1.5 cm^2^ YSL-30T carbon paper, uniformly spreading, obtaining electrode to be tested.

**Electrocatalysis experiments.** The electrocatalysis experiments were carried out in a separated flow cell with three chambers (Figure S16). At cathode chamber, carbon paper with the catalyst was used as working electrode (WE) and Hg/HgO electrode (with 1 M KOH used as the filling solution) was used as reference electrode (RE). A piece of Pt foil was used as counter electrode (CE) at the anode chamber. The gas chamber was separated from the cathode chamber by carbon paper and CO_2_ was diffused to the catholyte through a gas diffusion layer. During the CO_2_RR, the electrolysis occurs at the gas-liquid-solid three-phase interface. An anion-exchange membrane separated the cathode and anode chambers. All the electrodes and anion-exchange membrane were fixed and sealed by silicone pad, and their effective area for the electrolytic reaction was 1×1 cm^2^. In the experiments, all potentials were converted to the RHE reference scale using the relation E_RHE_ = E_Hg/HgO_ + 0.098 + 0.059 × pH and compensated with the solution resistance. 1 M KOH was used as the electrolyte, which was circulated through the cathodic and anodic chambers using peristaltic pumps at 10 mL·min^-1^. The flow rate of CO_2_ gas through the gas chamber was controlled to be 20 mL·min^-1^ using a digital gas flow controller. The linear sweep voltammetry (LSV) curves were obtained at a sweep rate of 20 mV·s^−1^.

The partial current densities of HCOOH (*J*_HCOOH_) were calculated as below:

*J*_HCOOH_ = *J*_total_ × FE

The turnover frequency (TOF) of HCOOH was calculated by the following equation:

TOF (h^-1^) = *J*_HCOOH_ × M × 3600 / (N × F × m × w%)

where N is the electron transfer number for HCOOH production, m is catalyst mass in the electrode (g), w% is metal Bi loading in the catalyst, and M is the atomic mass of Bi (208.98 g mol^-1^).

The Tafel slope was calculated according to the Tafel equation as following:

*η* = *b* log (*J*_HCOOH_ / *J*_0_)

Where *η* is overpotential, *b* is Tafel slope, and *J*_0_ is the exchange current density.

**Liquid-phase products analysis.** The liquid-phase products were analyzed using a Shimadzu LC-2050 high-performance liquid chromatograph. A mixture of 0.05% H_3_PO_4_ and acetonitrile (V/V = 95:5) was used as the mobile phase, with a flow rate of 1 mL/min. A C18 reverse-phase analytical column was employed, maintained at a column temperature of 30 °C. Prior to analysis, 1 mL of the electrolyte was neutralized with 1 mL of 0.5 M H_2_SO_4_, followed by filtration through a water-phase filter membrane to prepare the sample solution for measurement.

FE_HCOOH_ = (n × c × V_cathode_ × F ⁄ Q) × 100%

Where c is the concentration of the liquid-phase product which calculated based on the standard curve (Figure S17), V_cathode_ is the volume of cathode electrolyte, n is the number of transferred electrons and Q is the quantity of applied electric charges during the CO_2_ reduction.

**Gas-phase products analysis.** After electrolysis reaction, the gas products were analyzed by an on-line gas chromatograph (Techcomp, GC7980). The FID detector was employed to detect hydrocarbons and CO, while the TCD detector was used for H_2_ analysis, with argon as the carrier gas. Calibration curves were constructed using standard gases of known concentrations, which were diluted with argon to different concentrations, as shown in Figure S18. The FE of H_2_ and CO was calculated by using the concentrations (ppm) detected by the gas chromatograph as follows:

FE*_gas_* = (n × F × *p* V_gas_) / *J*_total_ × R × T × 100%

Where n is the number of transferred electrons, *p* is the atmospheric pressure, *T* = 298.15 K, and F is the Faraday constant (96485 C mol^-1^), V_gas_ is the volume of gas products which calculated based on the standard gas, *J*_total_ is the total current density, and R = 8.314 J mol^-1^ K^-1^ is the gas constant.

**In situ Raman spectra:** In situ Raman spectra were obtained on a LabRAM HR800 spectrometer, and using a standard three-electrode cell equipped with an observation quartz window. Carbon paper was used as the working electrode, with a Pt wire and an Ag/AgCl electrode (saturated with KCl) serving as the counter and reference electrodes, respectively. CO_2_ gas was bubbled at a constant rate to saturate the 0.5 M KHCO_3_ electrolyte. During in situ Raman experiments, a Renishaw Raman spectrometer (λ = 633 nm) was used to record spectra over a potential range of -0.3 V to -1.2 V vs RHE. The experimental data were baseline-corrected to facilitate trend comparison. We verified that similar FE of the products were achieved in both 1 M KOH and 0.5 M KHCO_3_ electrolytes, indicating that the reaction processes are likely consistent (Figure S26).

**In situ ATR-FTIR spectra:** In situ electrochemical Fourier-transform infrared spectroscopy (ATR-FTIR) measurements were performed using a Thermo Fisher Nicolet iS50 spectrometer equipped with liquid nitrogen cooling, and an MCT detector (Figure S27). In situ setup utilized the SPEC-I reflective infrared optical system from Shanghai Yuanfang Technology Co., Ltd. The working electrode was a hemispherical silicon prism coated with a gold layer to enhance the infrared signal, with a catalyst and carbon black mixture drop-cast onto its surface. The preparation steps are as follows: The silicon prism was polished with diamond paste and cleaned ultrasonically in deionized water. It was then immersed in a piranha solution (98% H_2_SO_4_: 30% H_2_O_2_, v/v = 3:1) for 2 hours, followed by chemical deposition of a gold layer on its surface. Finally, a catalyst and carbon black ink was drop-cast onto the gold-coated surface to form the working electrode. During the experiment, a custom-designed three-electrode electrochemical cell was used, with a platinum wire as the counter electrode and an Ag/AgCl (saturated KCl) electrode as the reference. The electrolyte was 0.5 M KHCO_3_, continuously saturated with CO_2_ by bubbling during the measurements. The applied potential was scanned between 0 V and -1.7 V vs RHE.

**4. Computational Methods**

First-principles calculations were performed to get better insight of the structural properties via the density function theory (DFT) with the Vienna ab initio Simulation Package (VASP). The projector augmented wave (PAW) method computed by the Kohn-Sham equations, and the generalized gradient approximation (GGA) with the Perdew-Burke-Ernzerholf (PEB) was used to treat the electronic structure of the system. The van der Waals interactions were added to the standard DFT description by DFT-D3. A 15 Å vacuum between the layers was considered to avoid the interaction between the periodically repeated structures. In this work, the cut-off energy for plane-wave expansion was set to be 400 eV, and a Monkhorst-Pack k-point mesh of 2 × 2 × 1 was adopted. During the entire calculations, the convergence tolerances were chosen to be 10^-5^ eV for the total energy, 0.05 eV Å^-1^ for the final forces on all ions. The charge distribution was researched by charge density difference, with the isosurface value of the differential charge density set to 0.0006 e/Å^3^ to ensure comparability of the images. The density of states (DOS) was computed by the Gaussian smearing method with a smearing width of 0.1 eV. Free energy diagrams were established using the computational hydrogen electrode (CHE) method. The Gibbs free energies were calculated at 298 K and 1 atm according to G = E + E_ZPE_ − TS, where E is the total energy, E_ZPE_ is the zero-point vibrational energy, T is the thermodynamic temperature, and S is the entropy.


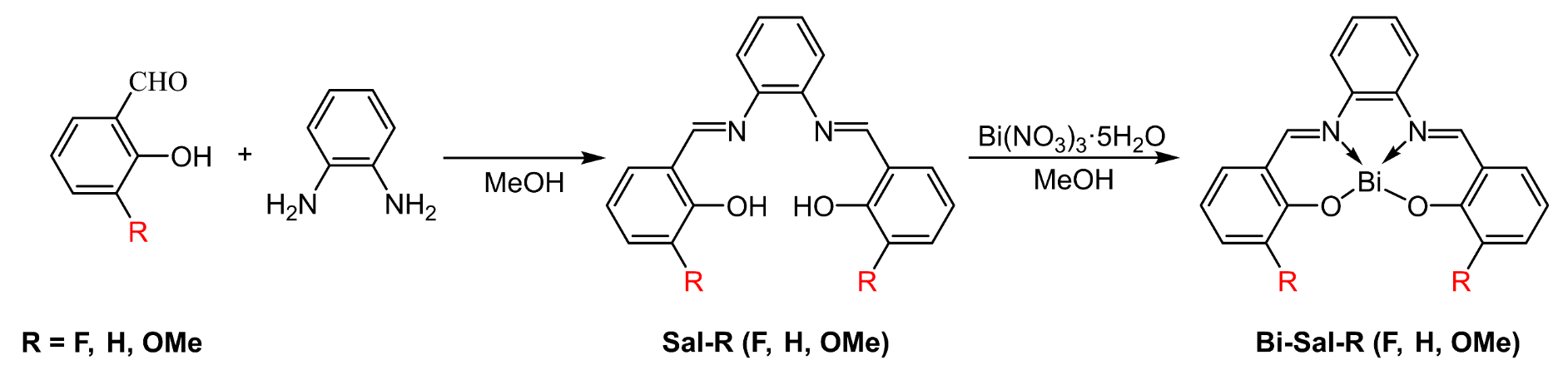


**Figure S1.** Synthetic scheme of Bi-Sal-F, Bi-Sal-H, and Bi-Sal-OMe samples.


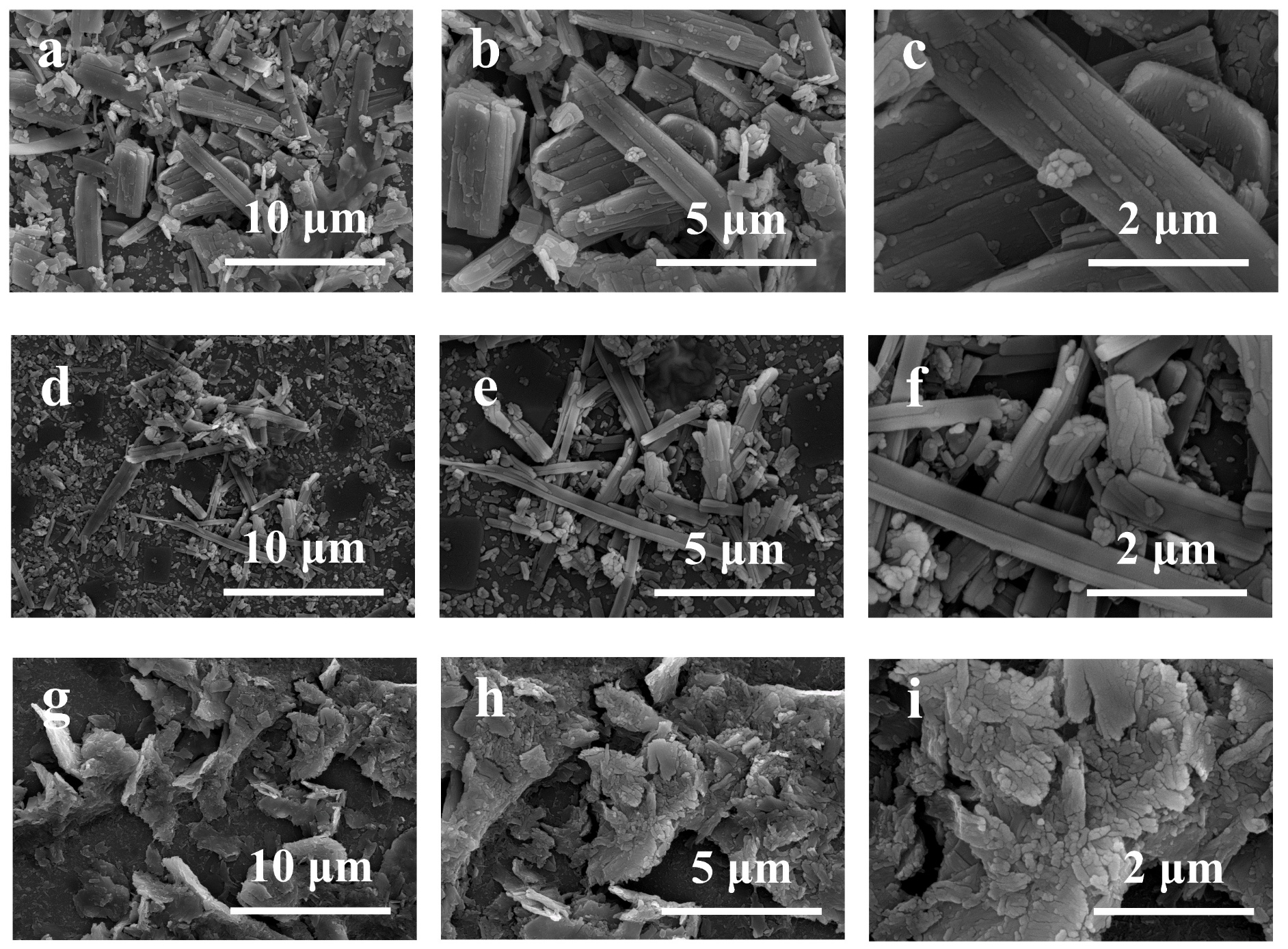


**Figure S2.** FESEM images of Bi-Sal-F (a-c), Bi-Sal-H (d-f), and Bi-Sal-OMe (g-i).


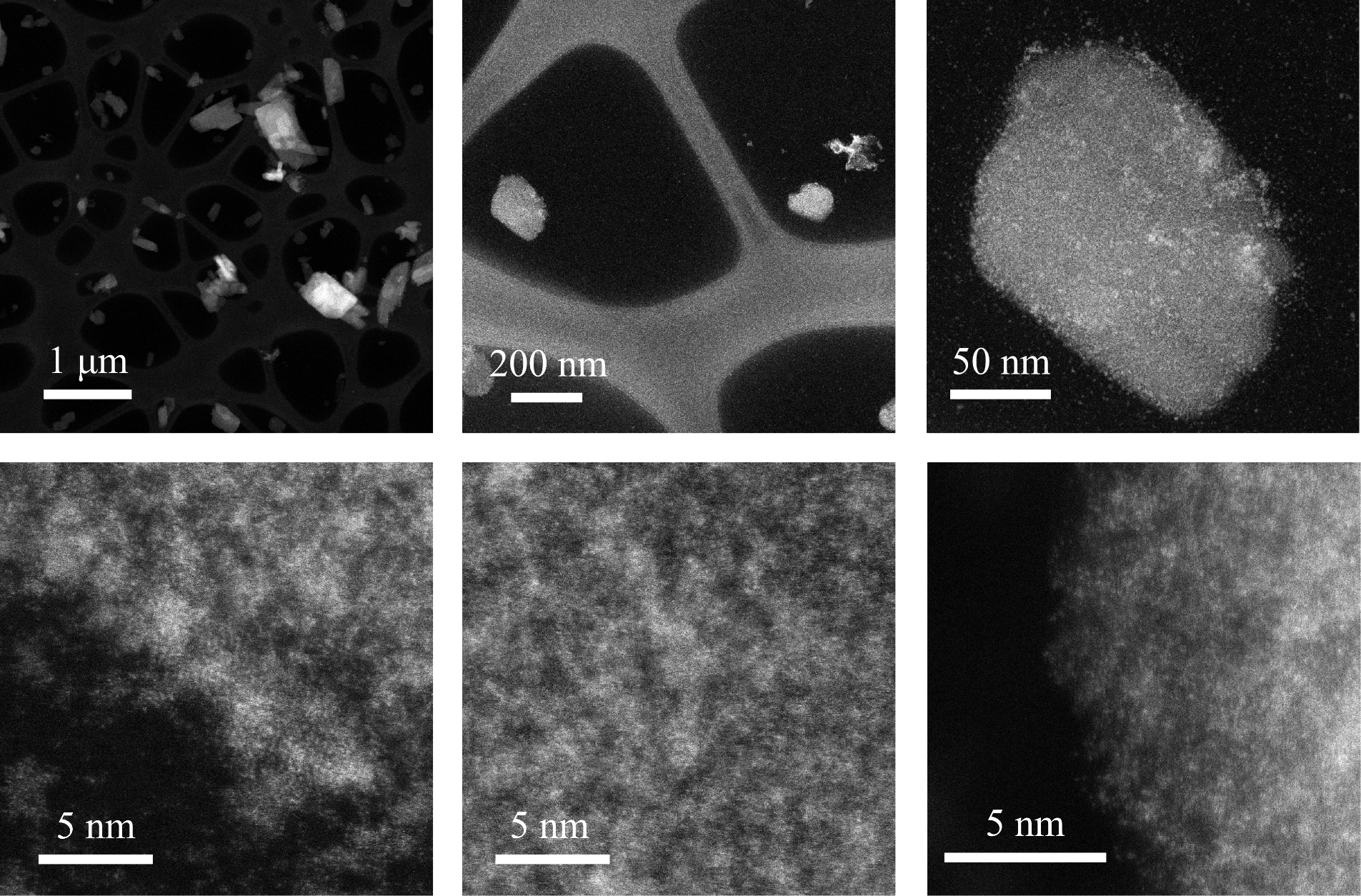


**Figure S3.** HAADF-STEM images of Bi-Sal-F.


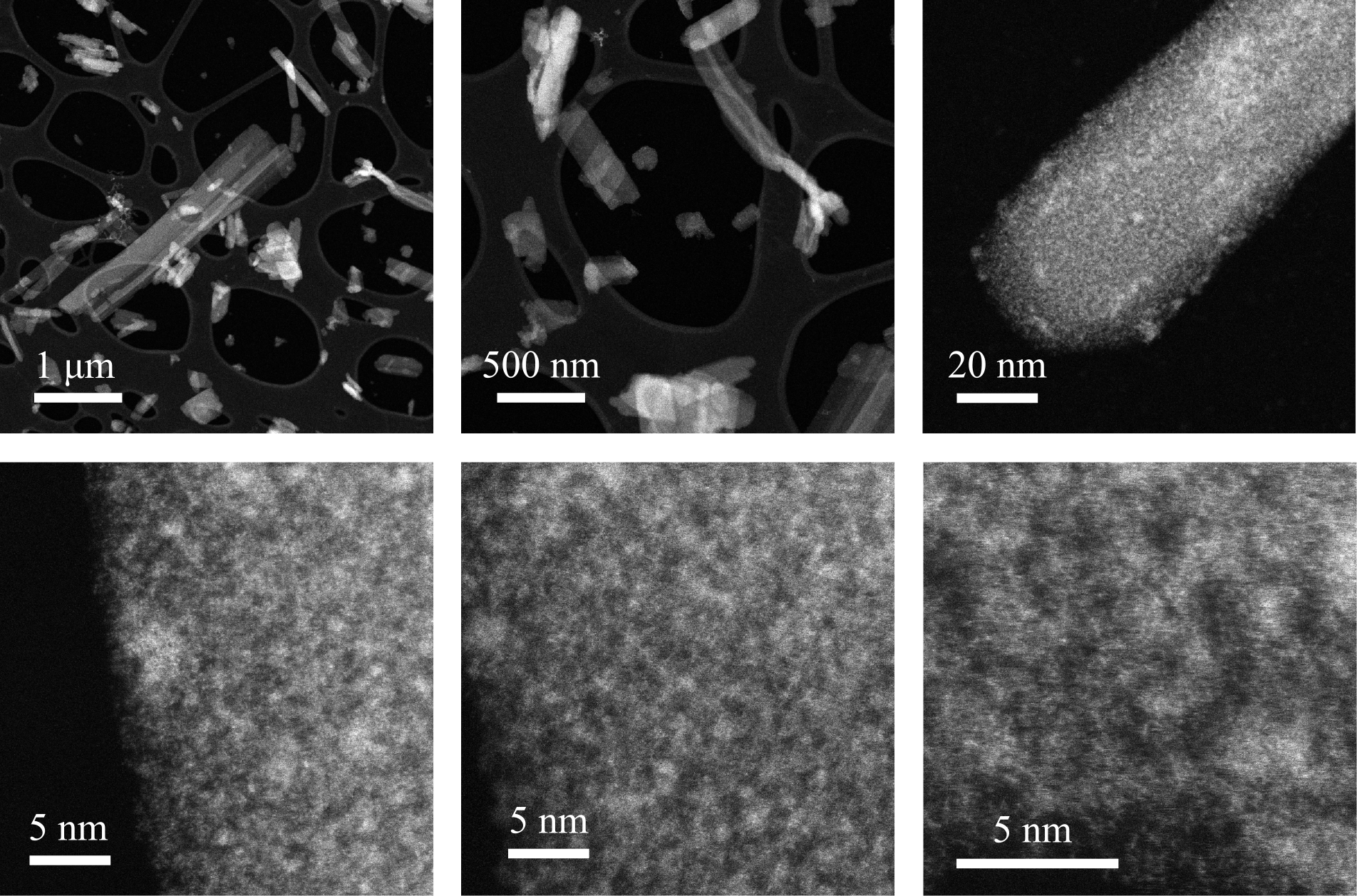


**Figure S4.** HAADF-STEM images of Bi-Sal-H.


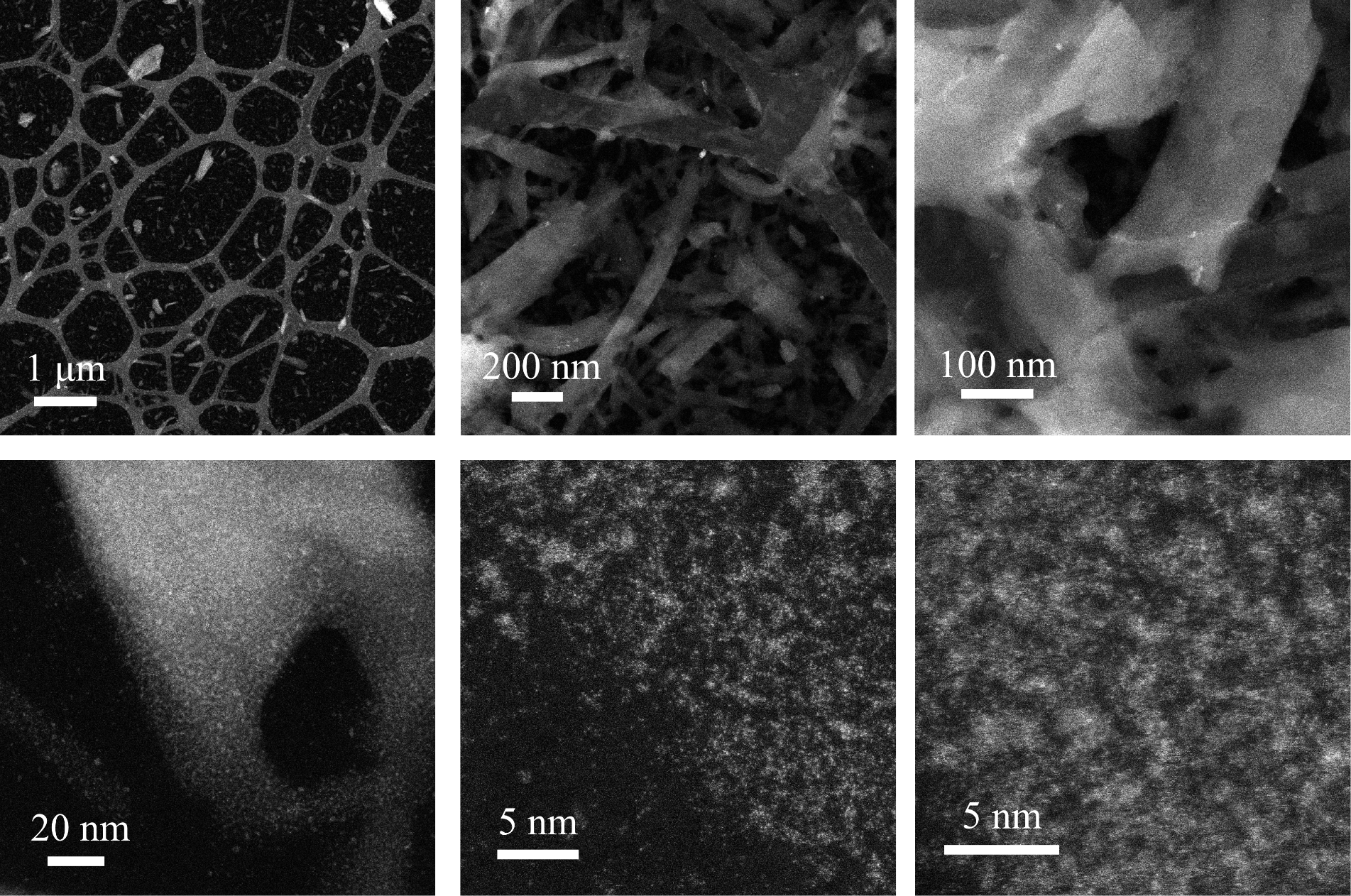


**Figure S5.** HAADF-STEM images of Bi-Sal-OMe.


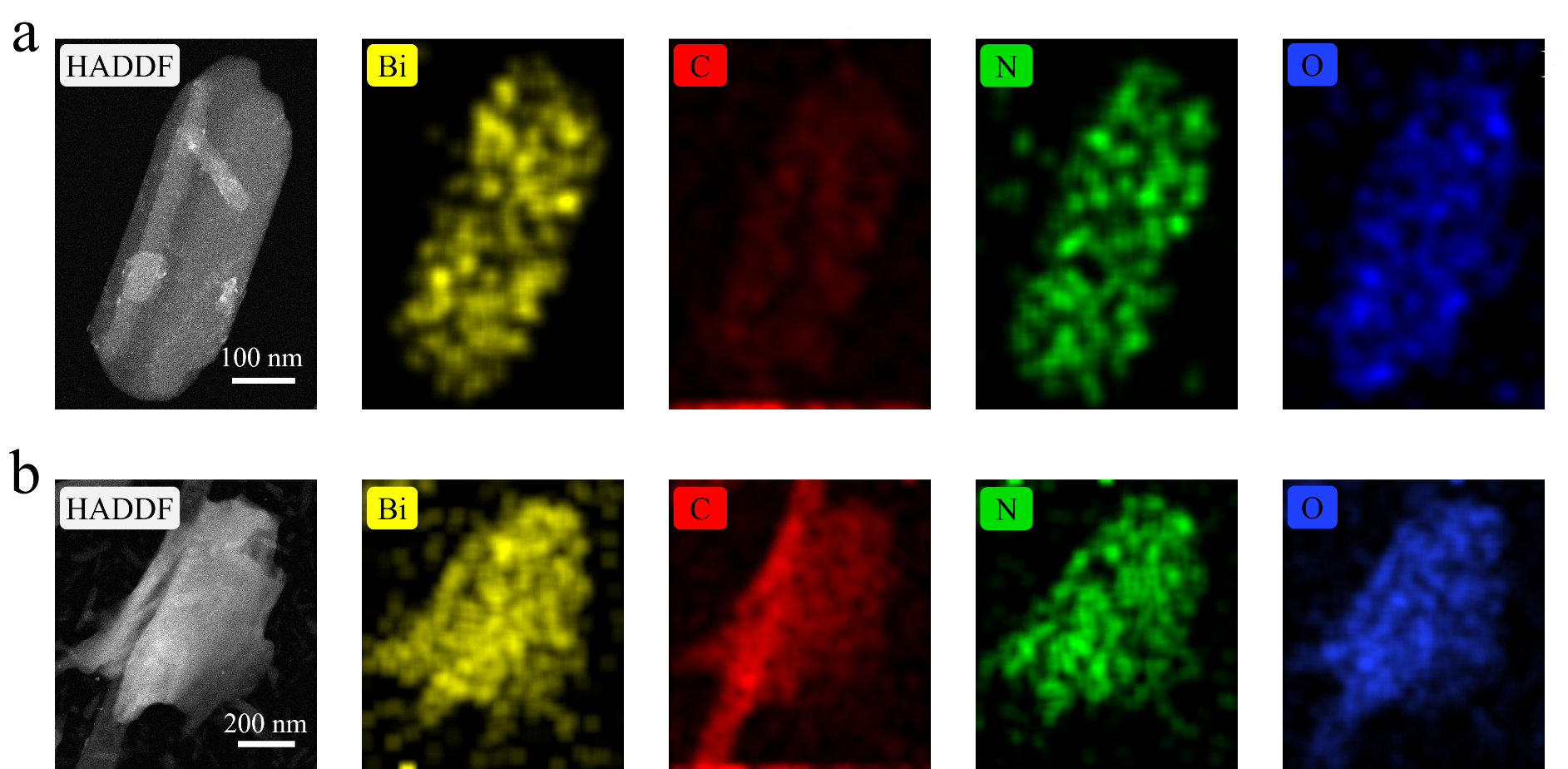


**Figure S6.** The HAADF-STEM image and the corresponding EDS mappings of Bi-Sal-H (a) and Bi-Sal-OMe (b).


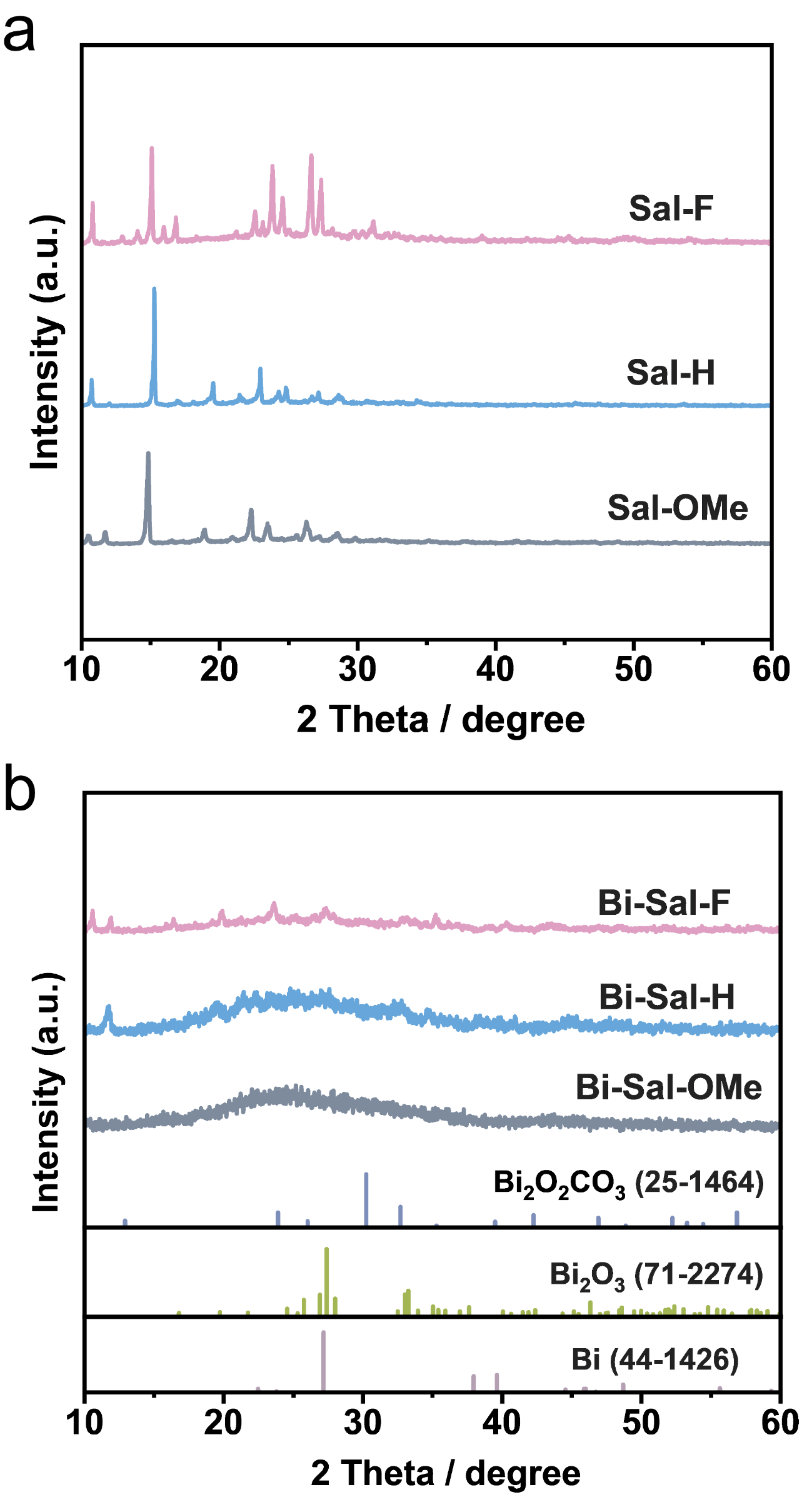


**Figure S7.** XRD spectra of (a) Sal-R and (b) Bi-Sal-R samples.^1-2^

**
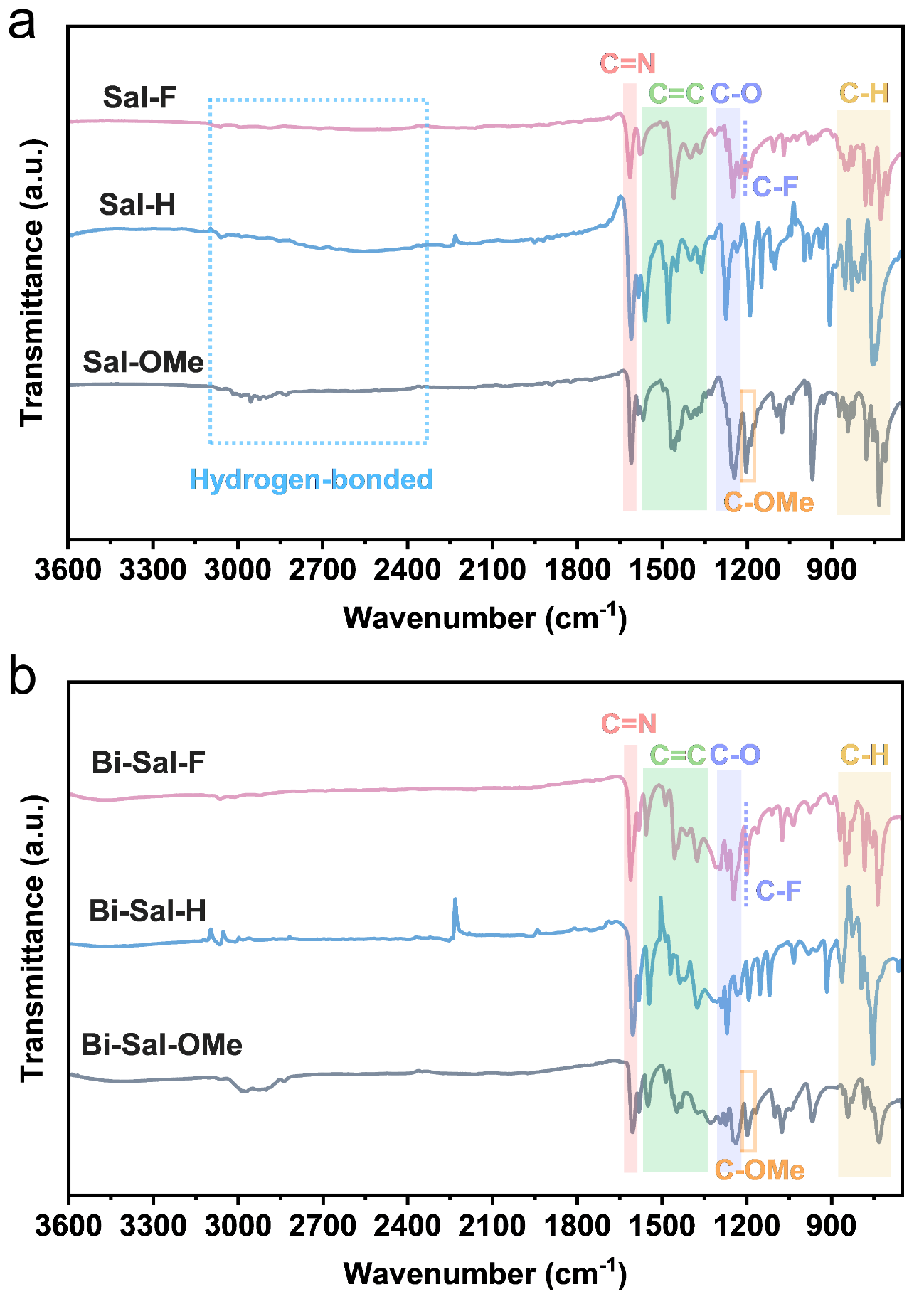
**

**Figure S8.** FTIR spectra of (a) Sal-R and (b) Bi-Sal-R samples.

The FTIR spectra of all samples confirmed the formation of Sal-R and Bi-Sal-R. The main characteristic peaks include: ~1613 cm^-1^ (HC=N), 1560-1360 cm^-1^ (aromatic C=C), ~1250 cm^-1^ (C-O), and 700-900 cm^-1^ (C-H of benzene ring).^3-4^ Additionally, the absorption peaks at ~1202 cm^-1^ (C-F) and ~1200 cm^-1^ (C-OMe) further support the structural features.^5-6^ The peak at ~2231 cm^-1^ arises from interference by atmospheric CO_2_ and is not an intrinsic feature of the compounds.^7^ Notably, Sal-R shows a broad absorption band at 2500-3300 cm^-1^, indicating intramolecular hydrogen bonding (O-H⋯N) and confirming its ordered structure.^8^ In contrast, this band nearly disappears in Bi-Sal-R, suggesting Bi replaces H, disrupting hydrogen bonds and forming an independent molecular complex.


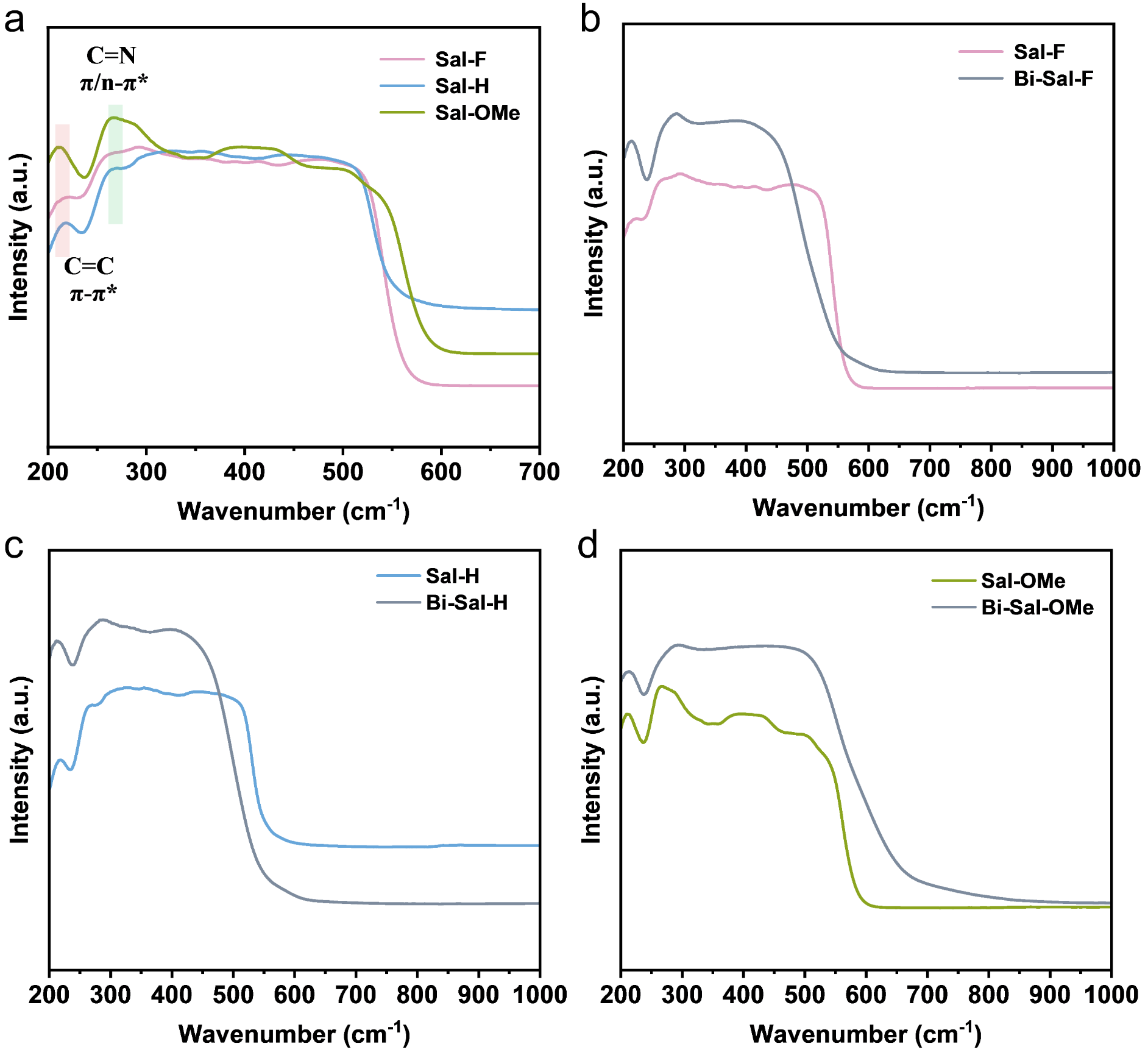


**Figure S9.** UV-vis spectra of (a) Sal-R and (b-d) Bi-Sal-R samples.

The Sal-R ligand exhibits distinct absorption peaks at ~217 nm and ~268 nm, corresponding to the π-π* transition of the benzene ring C=C bond and the n-π* transition of the C=N bond, respectively.^5^ The introduction of Bi^3+^ significantly enhances the UV absorption intensity without altering the peak profile of Sal-R, suggesting that Bi may coordinate with Sal-R.^9^


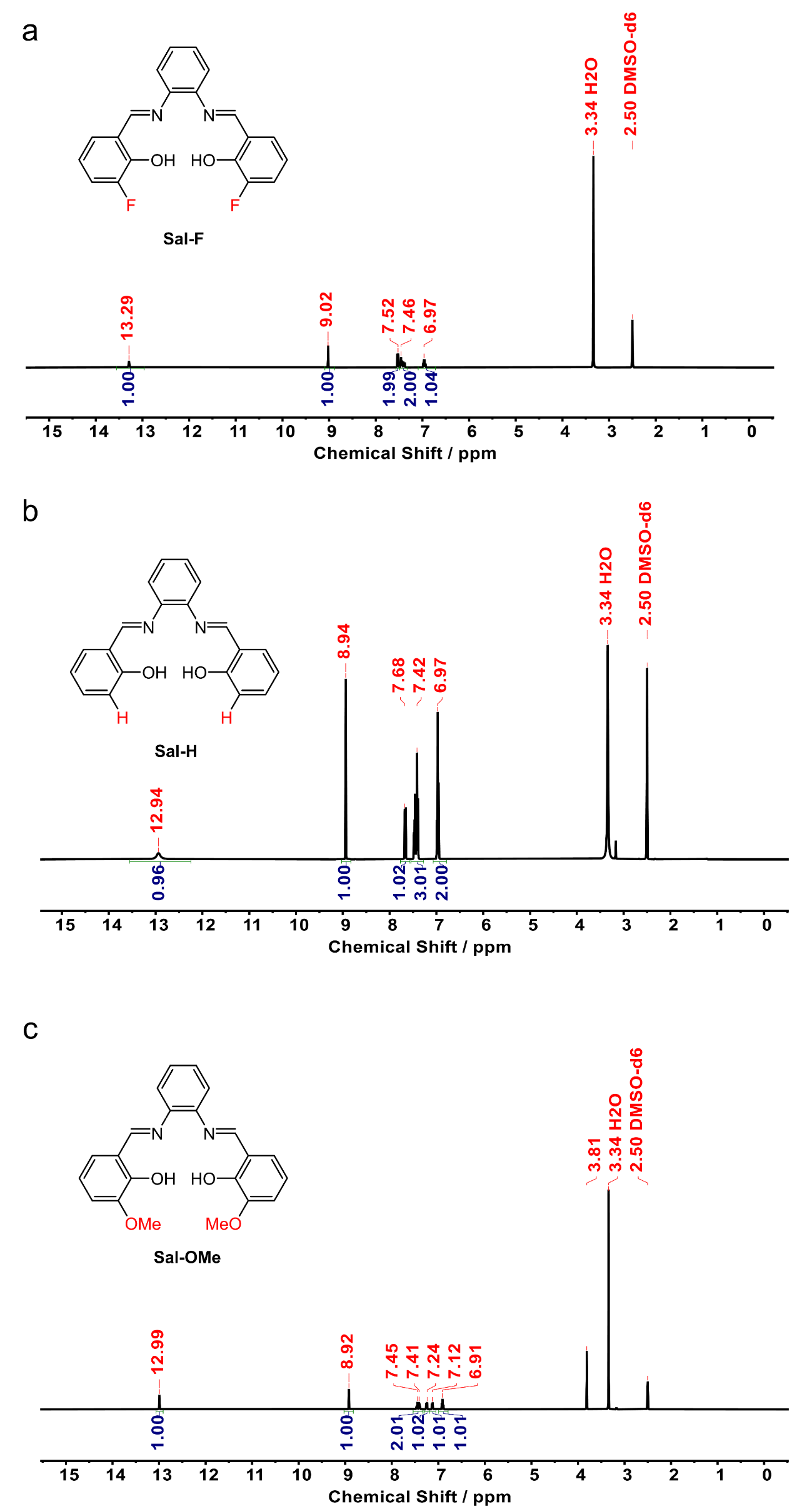


**Figure S10.** ^1^H NMR (in DMSO-d_6_) spectrum of Sal-R (a-c). In the graph, the red font represents the peak positions, while the blue font indicates the peak areas.

The chemical shifts are observed at ~13 ppm (-OH), ~9 ppm (-CH=N-), and 6-8 ppm (benzene ring hydrogens). The comparable peak areas of -OH and -CH=N- confirm the successful synthesis.


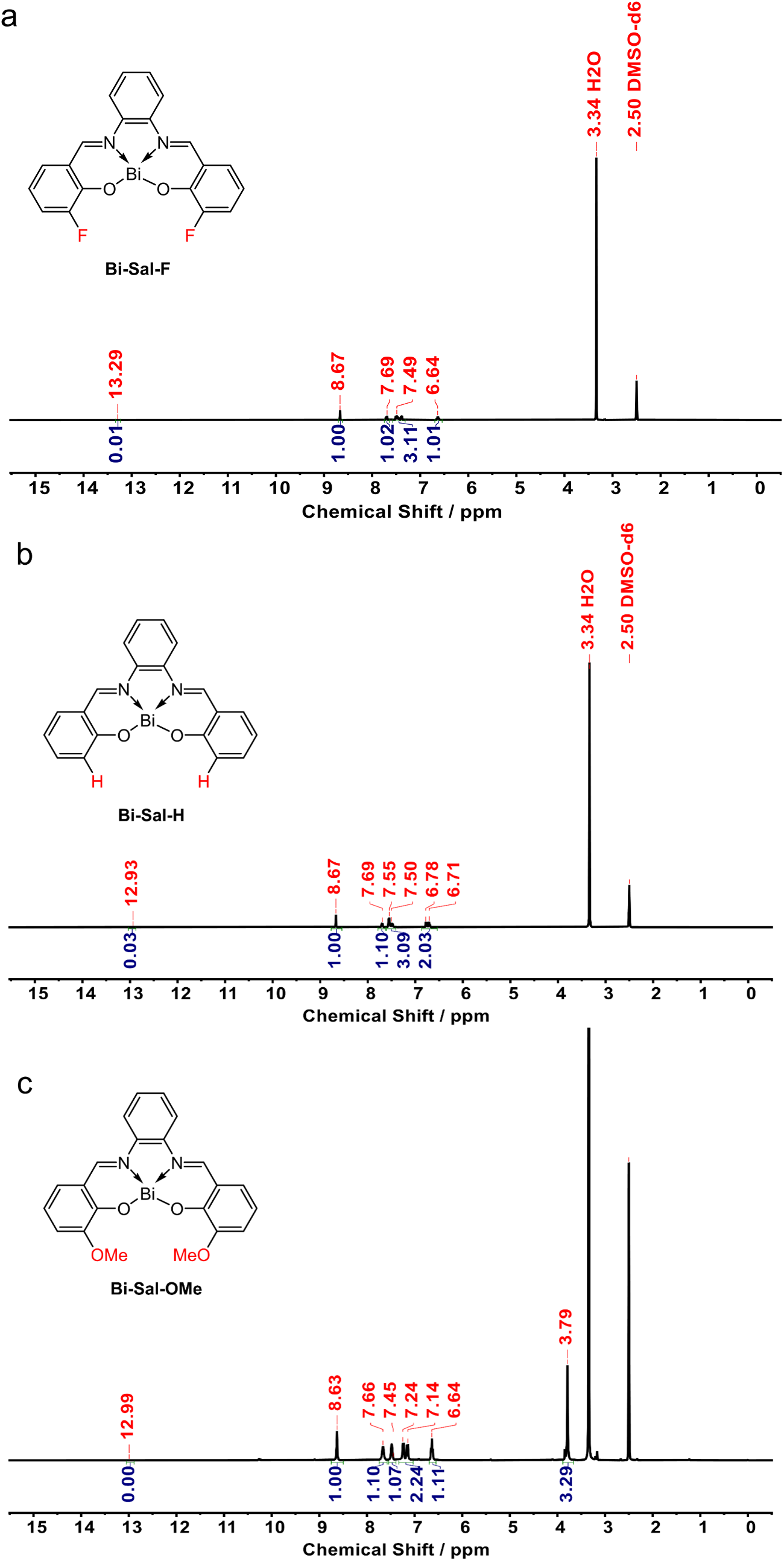


**Figure S11.** ^1^H NMR (in DMSO-d_6_) spectrum of Bi-Sal-R (a-c). In the graph, the red font represents the peak positions, while the blue font indicates the peak areas.

The absence of -OH signals indicate successful replacement by Bi atoms, confirming Bi-Sal-R formation.

**
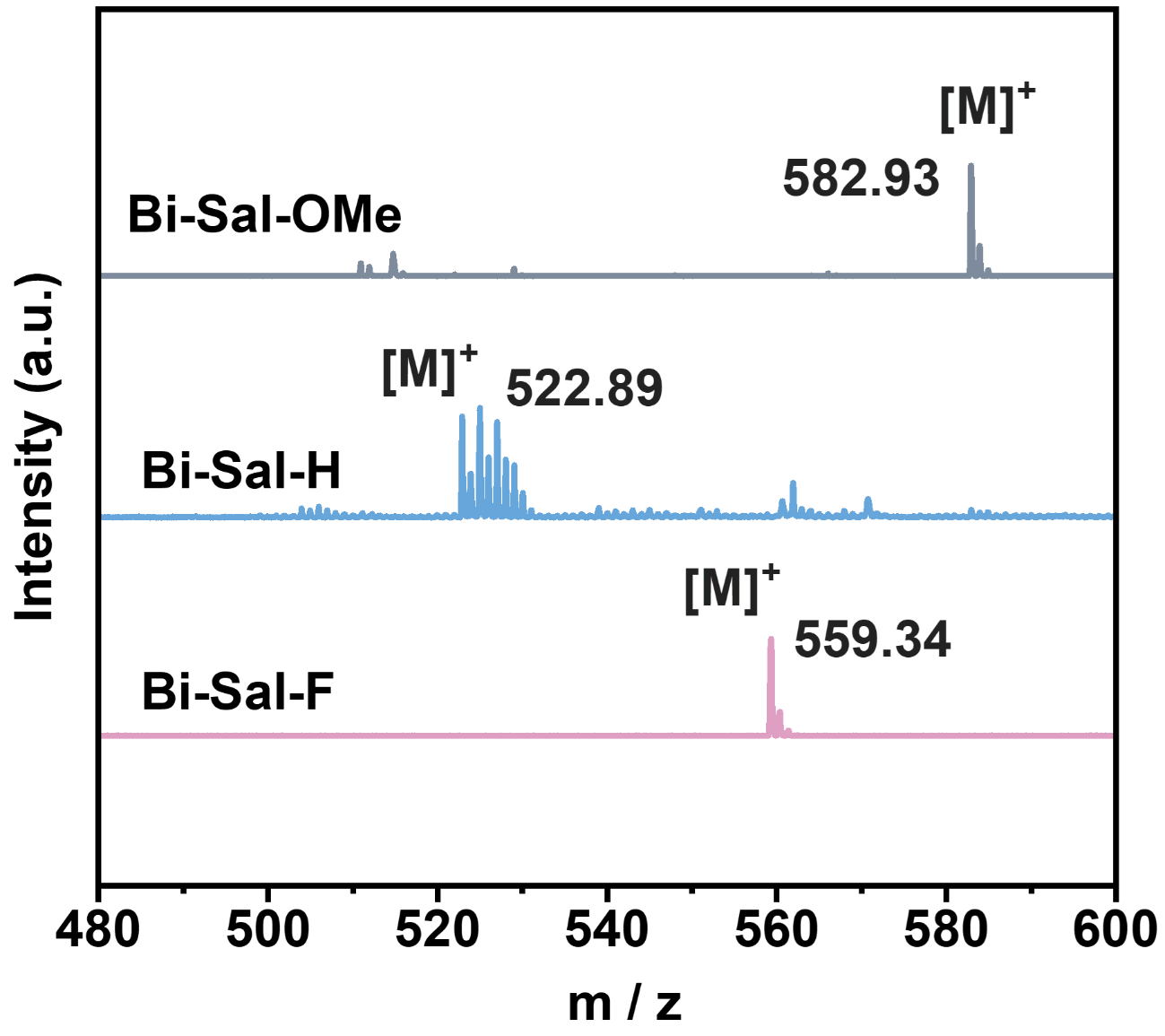
**

**Figure S12.** MALDI-TOF MS spectra of Bi-Sal-F, Bi-Sal-H, and Bi-Sal-OMe.


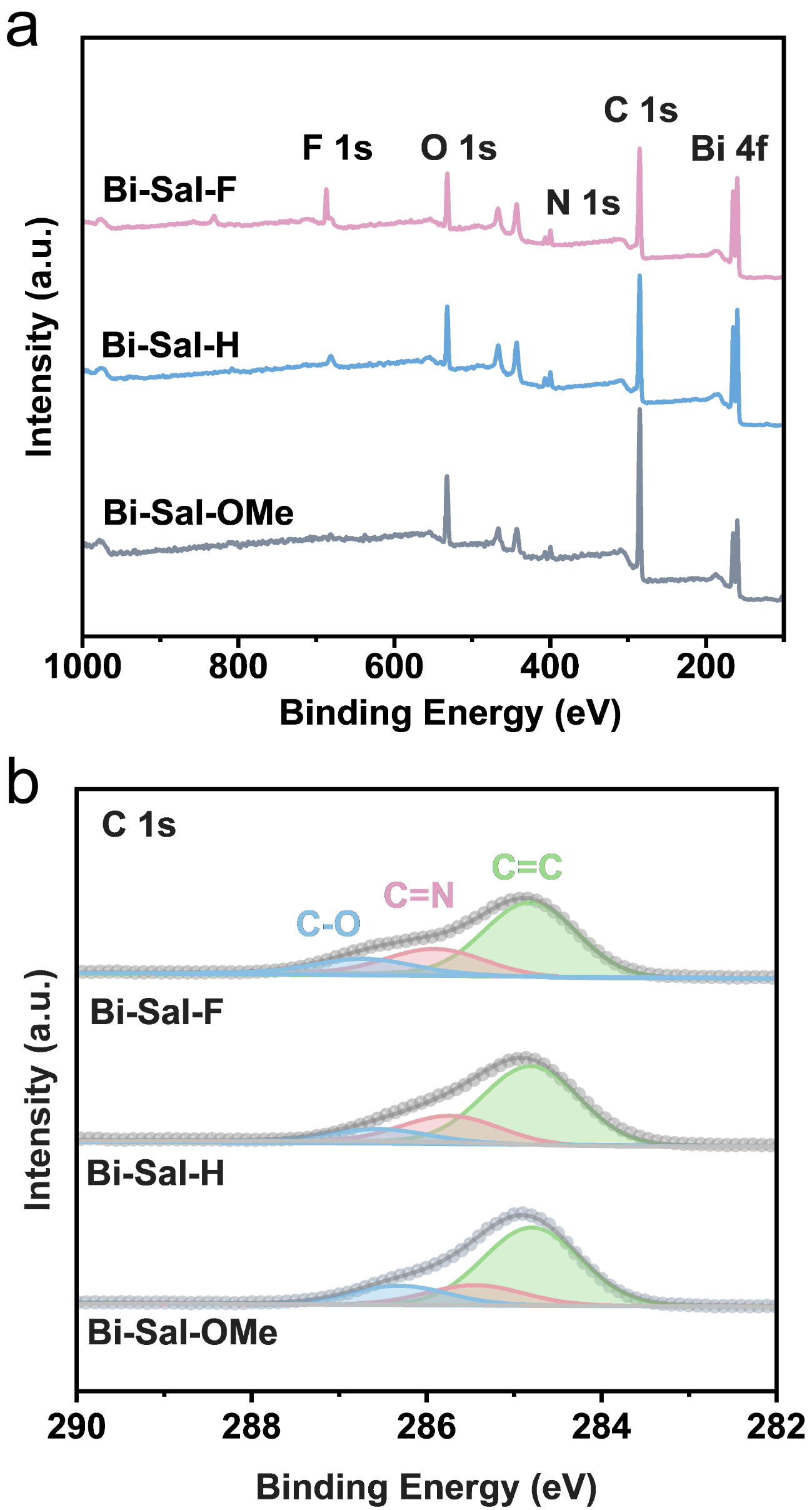


**Figure S13.** (a) XPS spectra of Bi-Sal-R samples. (b) The high-resolution C 1s XPS spectra of Bi-Sal-R samples.^10-11^

**
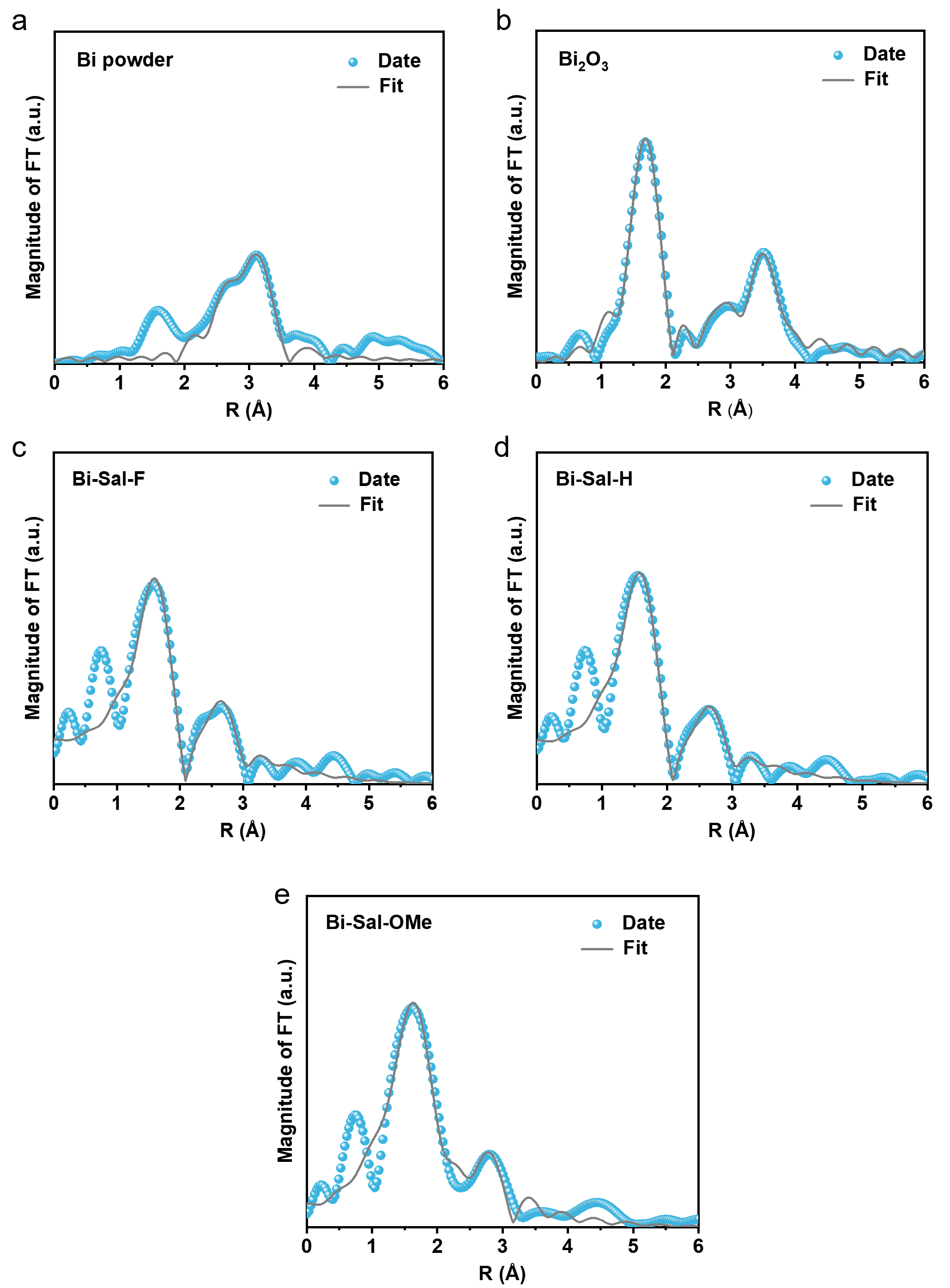
**

**Figure S14.** Bi R-space EXAFS (points) and curve fit (line) for various samples. The data are not phase-corrected.


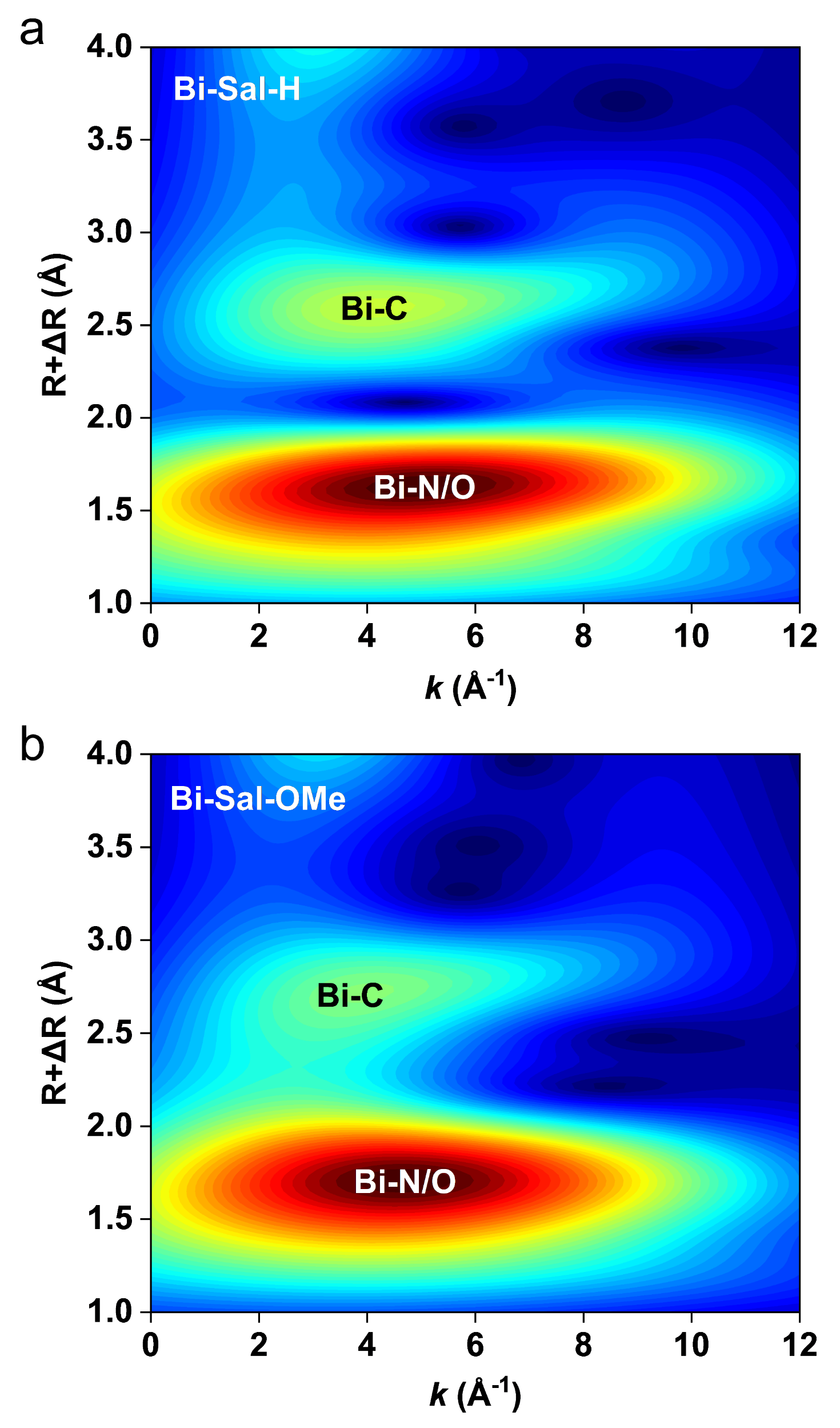


**Figure S15.** Wavelet transform of the k^3^-weighted EXAFS for the Bi *L*_3_-edge in Bi-Sal-H (a) and Bi-Sal-OMe (b).


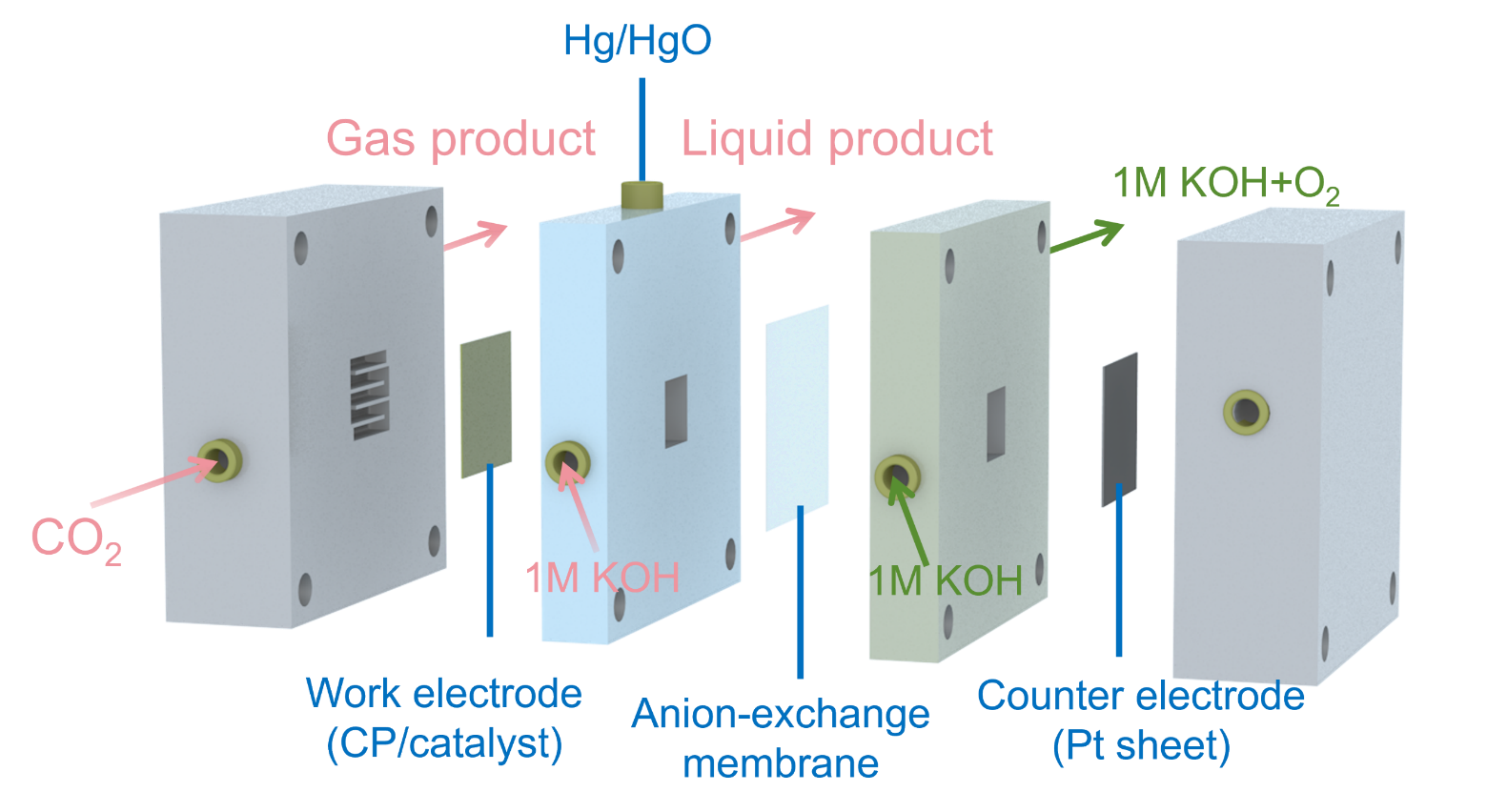


**Figure S16.** Schematic of the electrocatalytic flow cell.


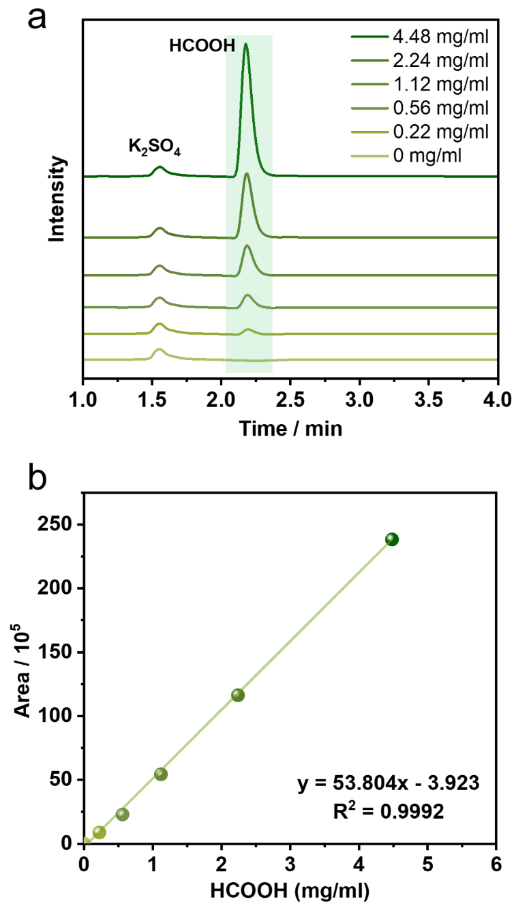


**Figure S17.** Peak areas (a) and standard curve (b) for HCOOH measured by liquid chromatography.

The calibration curve for HCOOH was established using HPLC with external standard method. HCOOH standard (99.5%) was dissolved in 1 M KOH solution and serially diluted to prepare standard solutions ranging from 0 to 4.485 mg/mL. After neutralization with an equal volume of 0.5 M H_2_SO_4_, the solutions were filtered through 0.22 μm membranes for HPLC analysis. The retention time of HCOOH was 2.2 min. The standard curve was obtained by linear regression of the integrated peak areas versus concentrations, showing good linearity (R^2^ = 0.9992). Detailed analytical methods and instrument parameters are provided in the Electrocatalysis experiments section of SI.


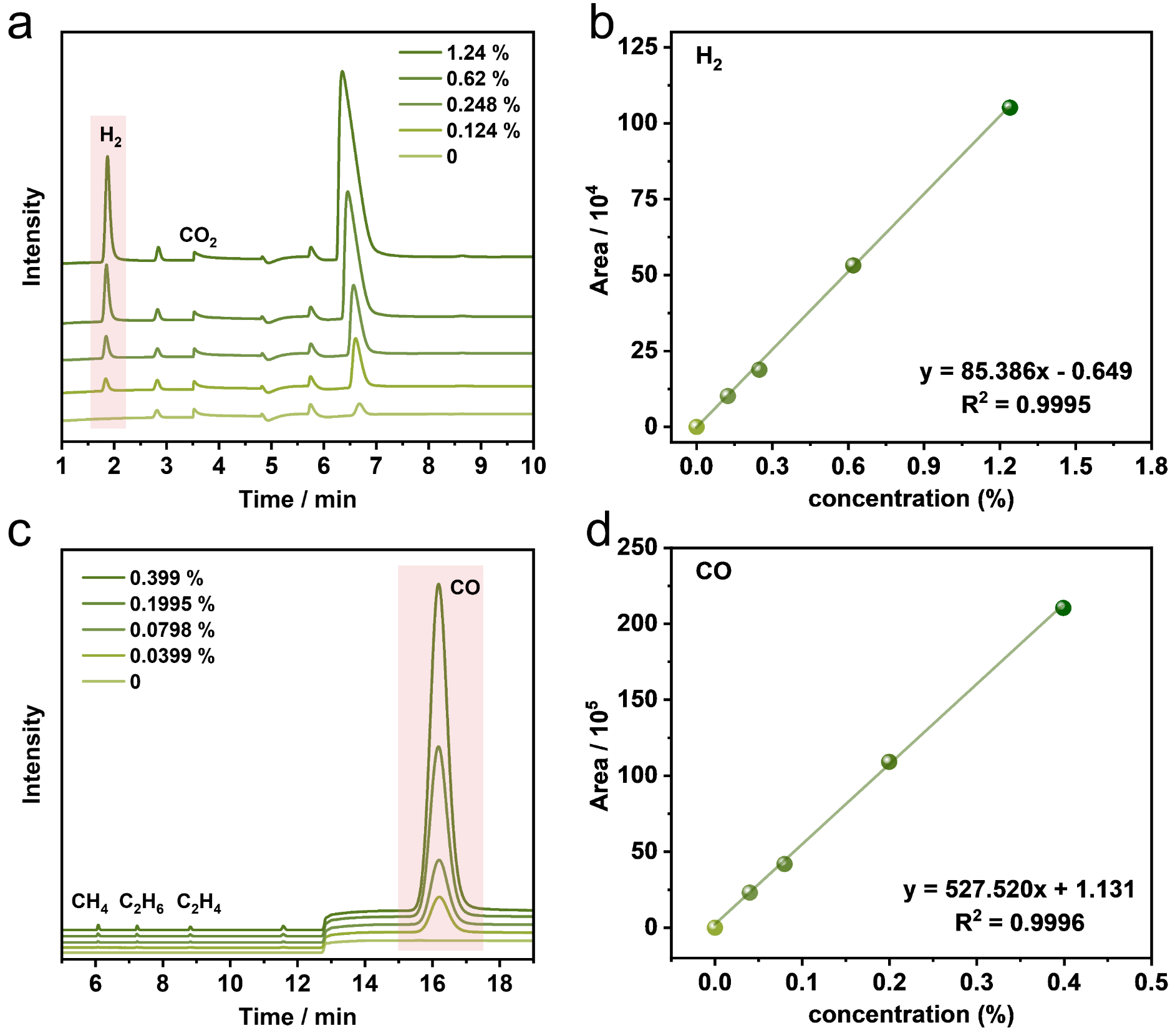


**Figure S18.** Peak areas (a, c) and standard curve (b, d) for H_2_ and CO measured by gas chromatography.

The calibration curves for H_2_ and CO were established using gas chromatography with external standard method. H_2_ was detected by TCD with a retention time of 1.7 min, while CO was analyzed by FID showing a retention time of 16.2 min. Standard gases containing H_2_ (1.24%) and CO (0.399%) were diluted with argon to generate concentration series ranging from 0-1.24% and 0-0.399%, respectively. Linear regression of peak area versus concentration yielded calibration curves with correlation coefficients of R^2^=0.9995 for H_2_ and R^2^=0.9996 for CO. Detailed analytical methods and instrument parameters are provided in the Electrocatalysis experiments section of the SI.


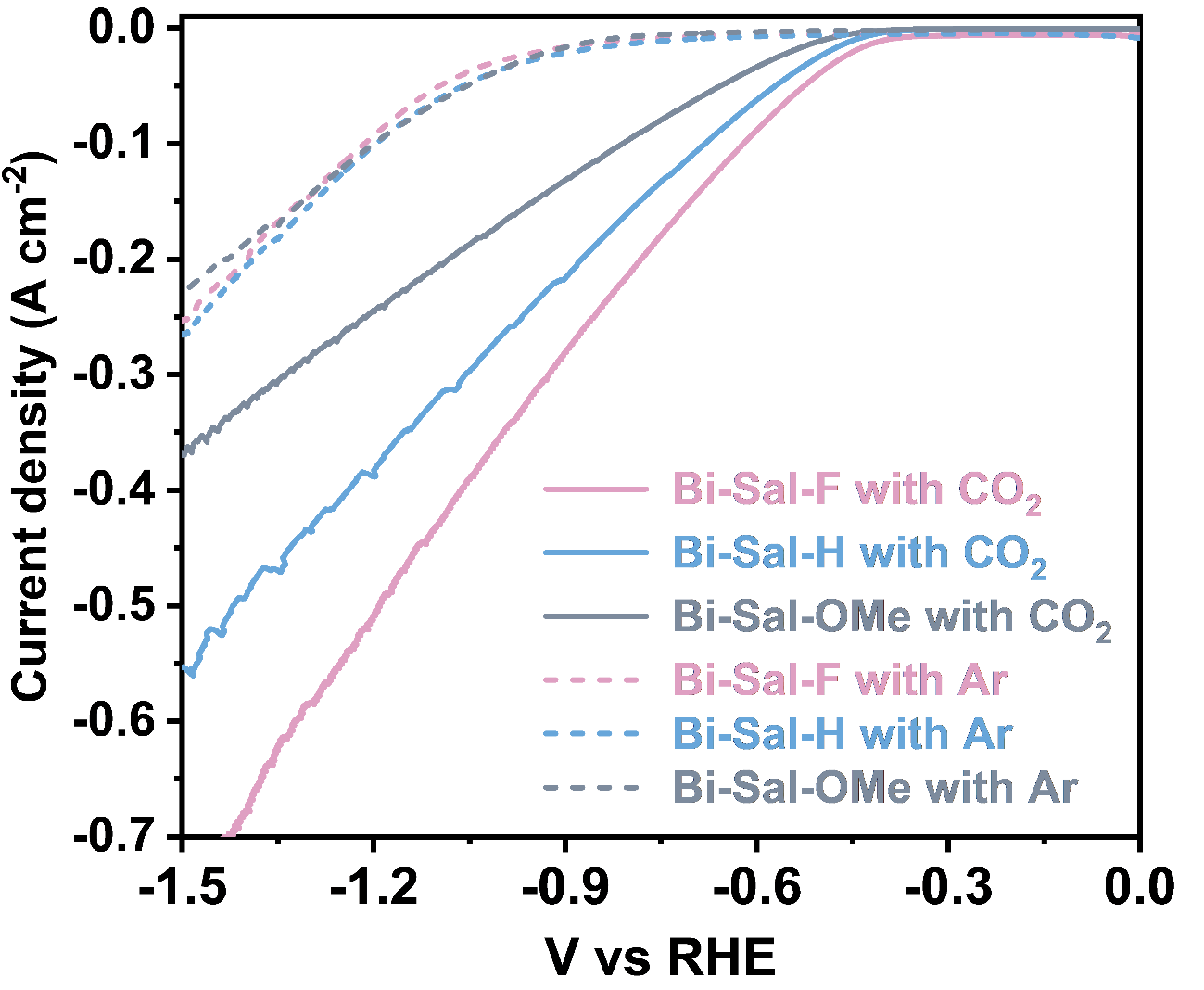


**Figure S19.** LSV curves in a flow cell under Ar and CO_2_ gas conditions.


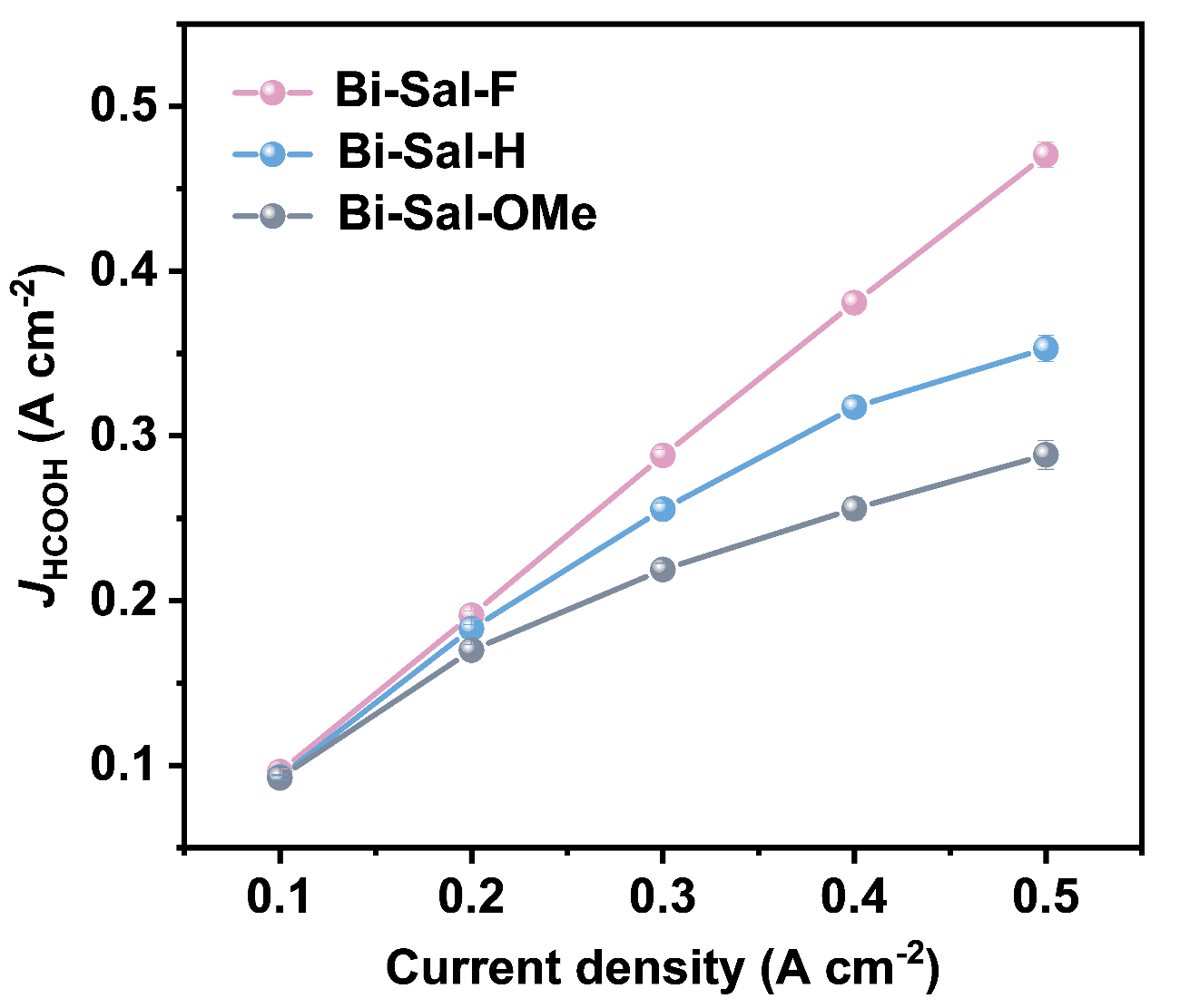


**Figure S20.** Partial current density of HCOOH under different current densities.


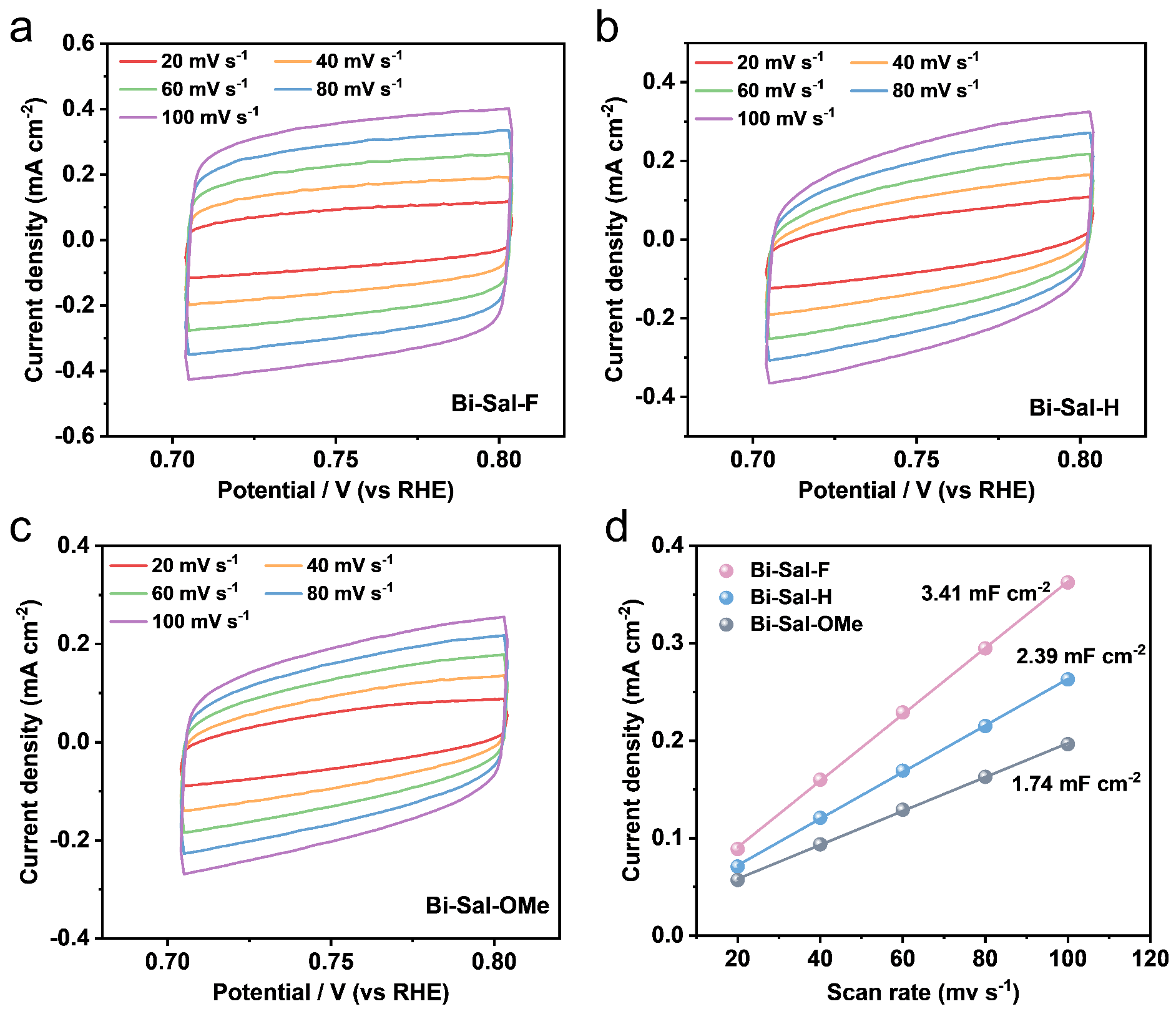


**Figure S21.** CV curves of (a) Bi-Sal-F, (b) Bi-Sal-H, (c) Bi-Sal-OMe in the double-layer region at scan rates of 20, 40, 60, 80 and 100 mV s^−1^ in CO_2_-saturated 1 M KOH in flow Cell. (d) The corresponding C_dl_ of different catalysts obtained by CV curves at different scan rates.


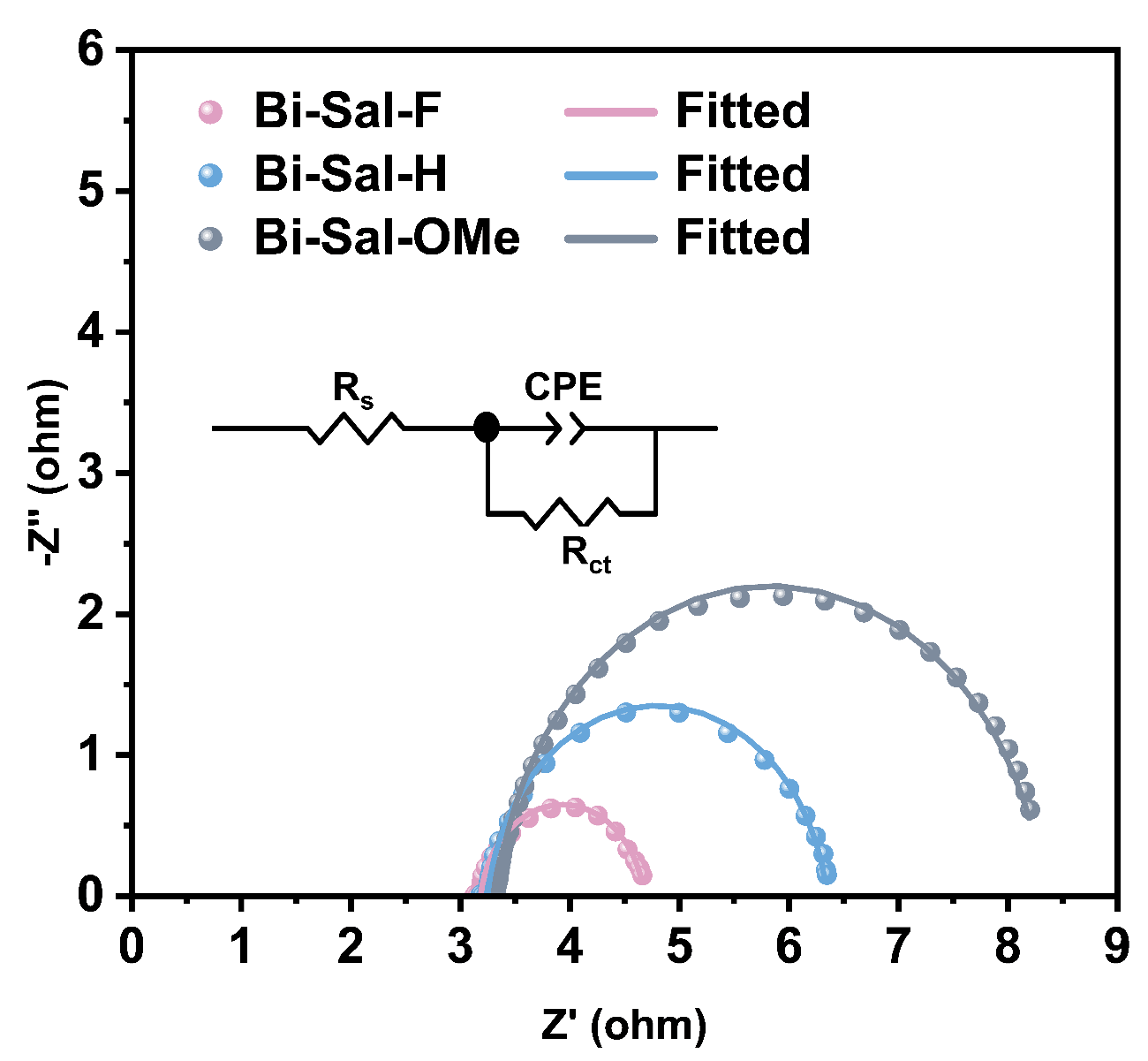


**Figure S22.** Nyquist plots of Bi-Sal-R measured at −0.6 V vs RHE in a CO_2_-fed flow cell with 1 M KOH electrolyte. The inset displays the equivalent circuit, where R_s_ is the solution resistance and R_ct_ is the charge transfer resistance. CPE is the constant phase element.


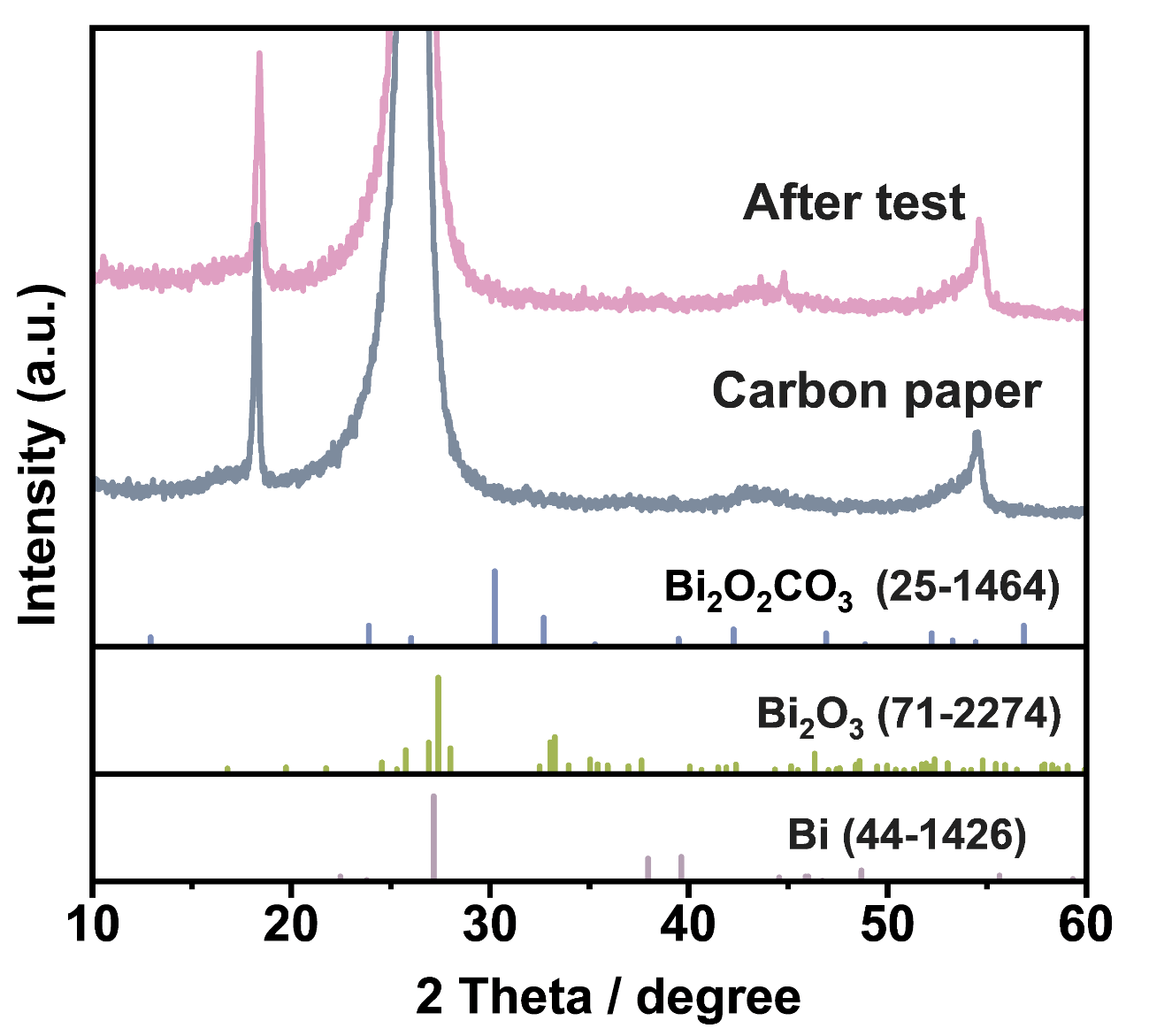


**Figure S23.** XRD patterns of the Bi-Sal-F catalyst after prolonged electrolysis. The spectrum of the carbon paper shown here does not include the Bi-Sal-F catalyst.


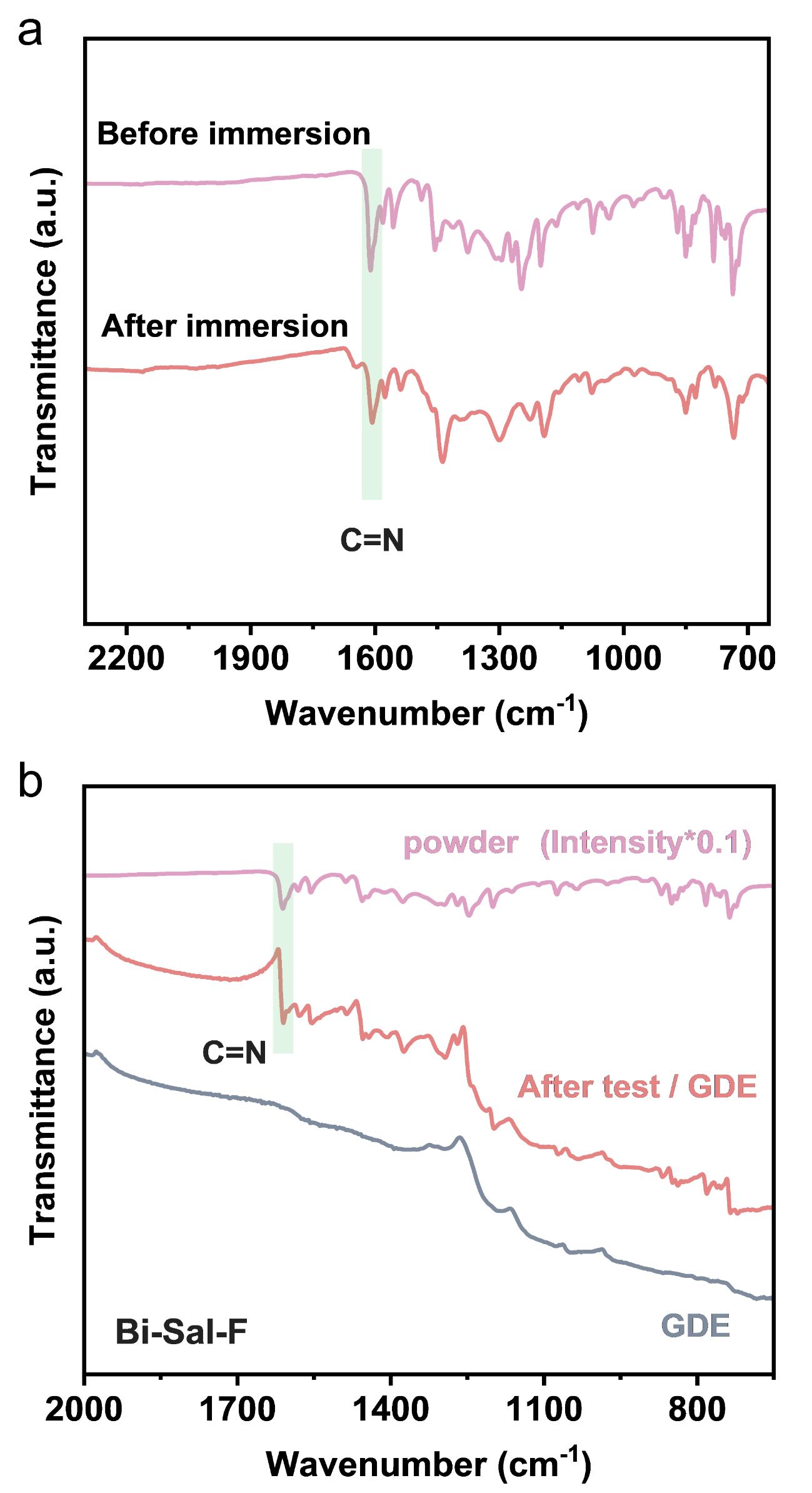


**Figure S24.** (a) FTIR spectra of the Bi-Sal-F sample before and after immersion in 1 M KOH. (b) FTIR spectra of the Bi-Sal-F sample after electrolysis on a gas diffusion electrode (GDE).


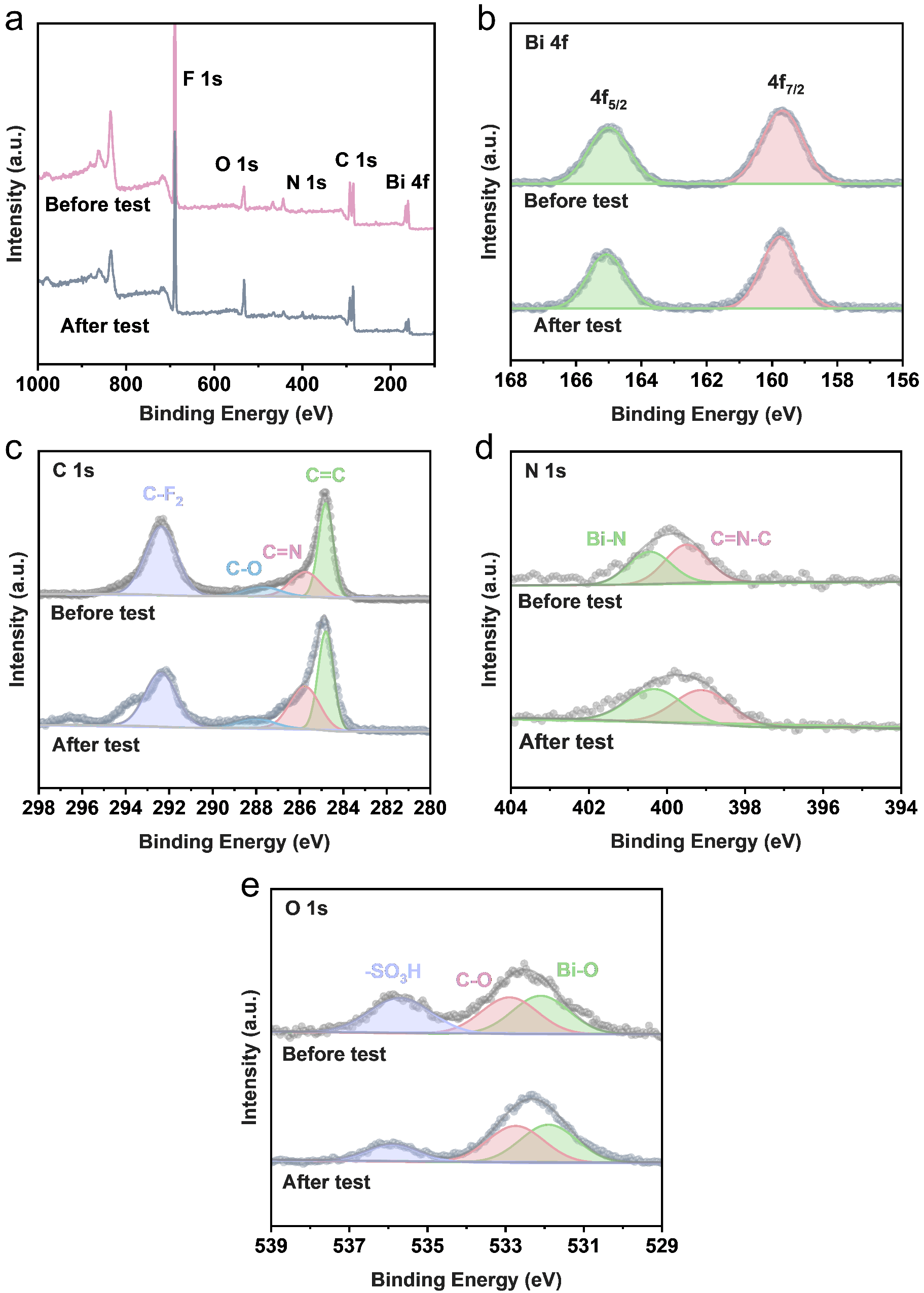


**Figure S25.** XPS patterns of the Bi-Sal-R catalyst after long-term electrolysis.

Notably, the C 1s peak at 292.4 eV corresponds to C-F_2_, while the newly observed O 1s peak at 535.7 eV originates from –SO_3_H, both of which are attributed to PTFE in the GDE and Nafion in the catalyst ink.^12-14^


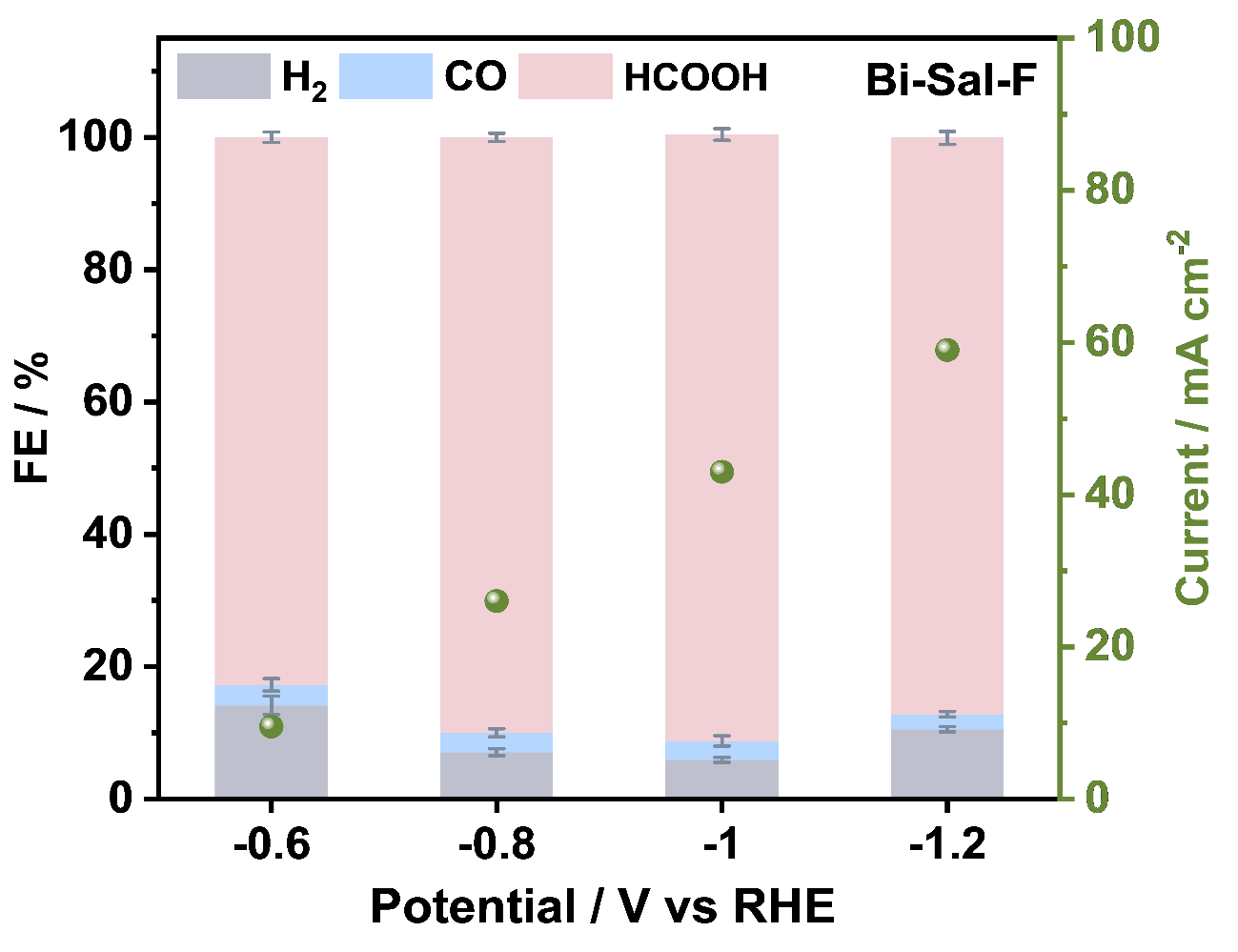


**Figure S26.** CO_2_RR performance at 0.5M KHCO_3_ in H-type cell.


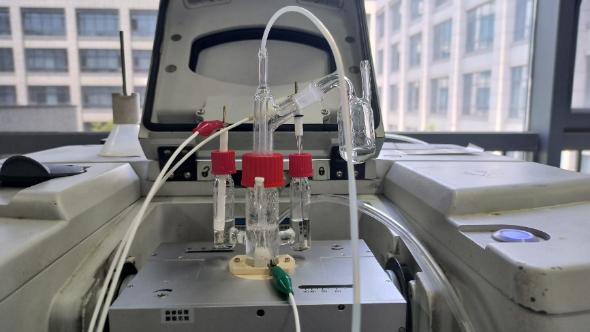


**Figure S27.** Schematic diagram of in situ ATR-FTIR.


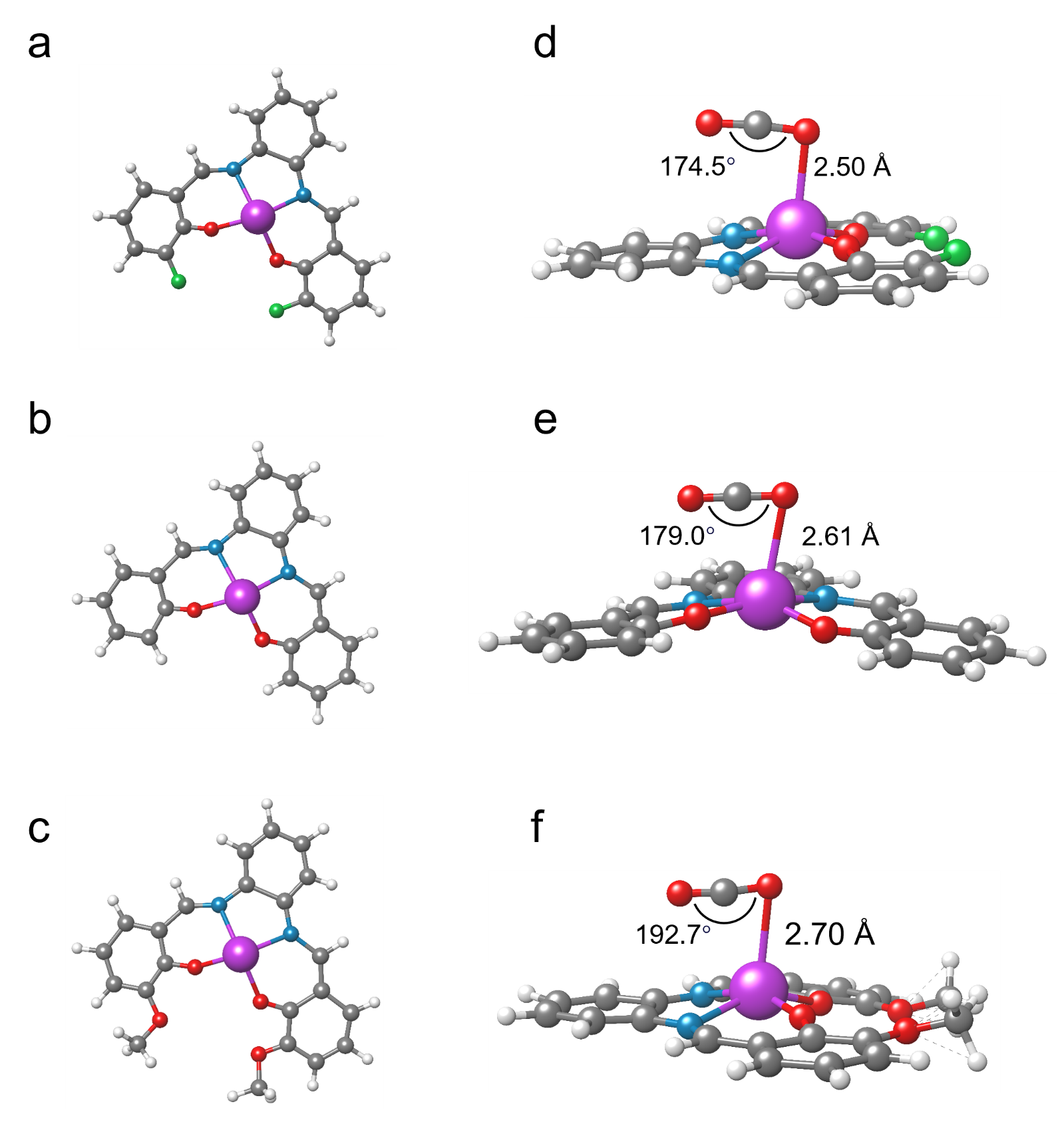


**Figure S28.** Optimized structural models of Bi-Sal-R catalysts and their corresponding CO₂ adsorption configurations. (a, d) Bi-Sal-F, (b, e) Bi-Sal-H, (c, f) Bi-Sal-OMe.


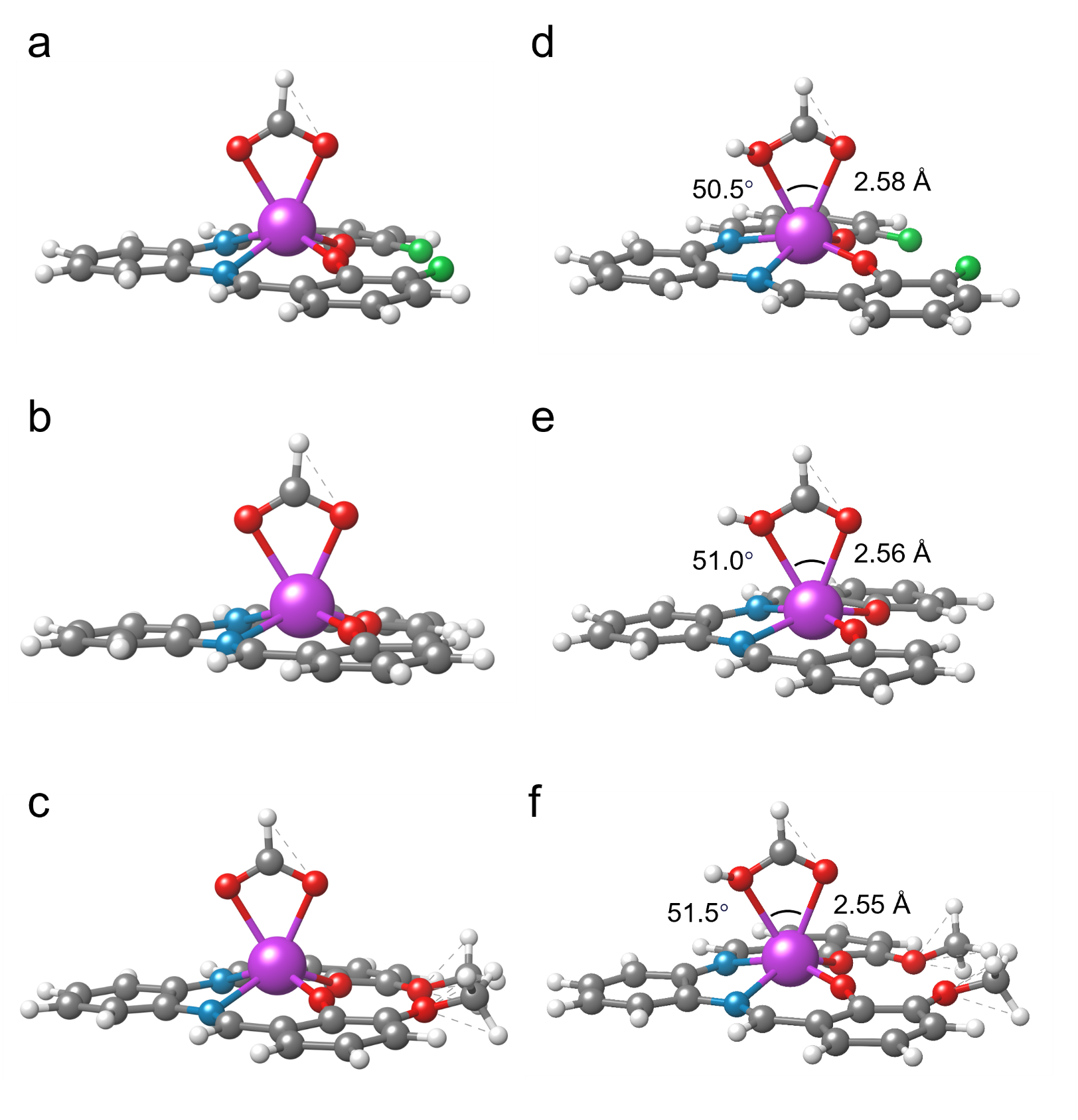


**Figure S29.** Optimized structural models of Bi-Sal-R catalysts with adsorbed *OCHO and *HCOOH intermediates during CO₂RR. (a, d) Bi-Sal-F, (b, e) Bi-Sal-H, (c, f) Bi-Sal-OMe.


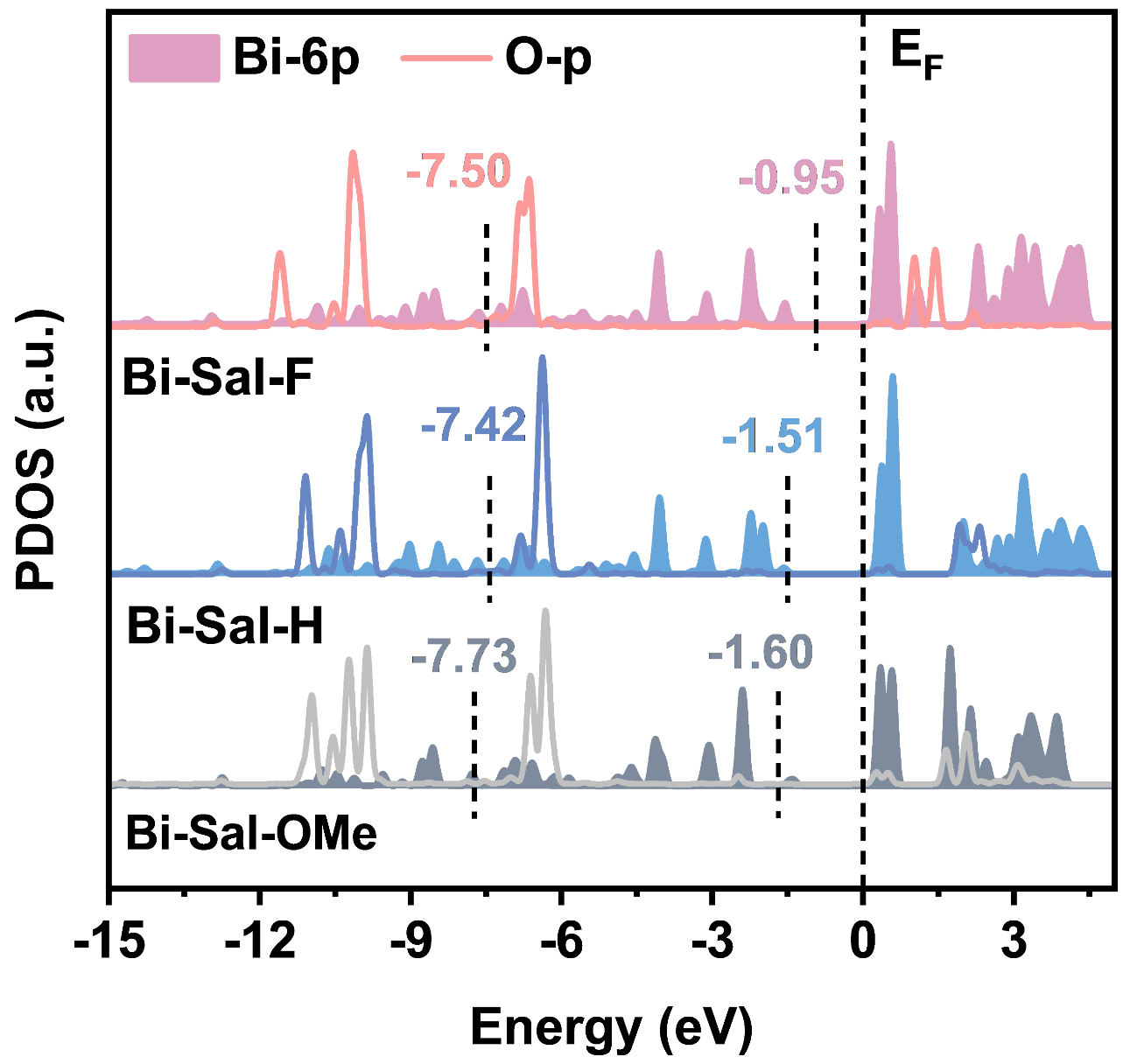


**Figure S30.** PDOS for Bi 6p and O-p orbitals.

**
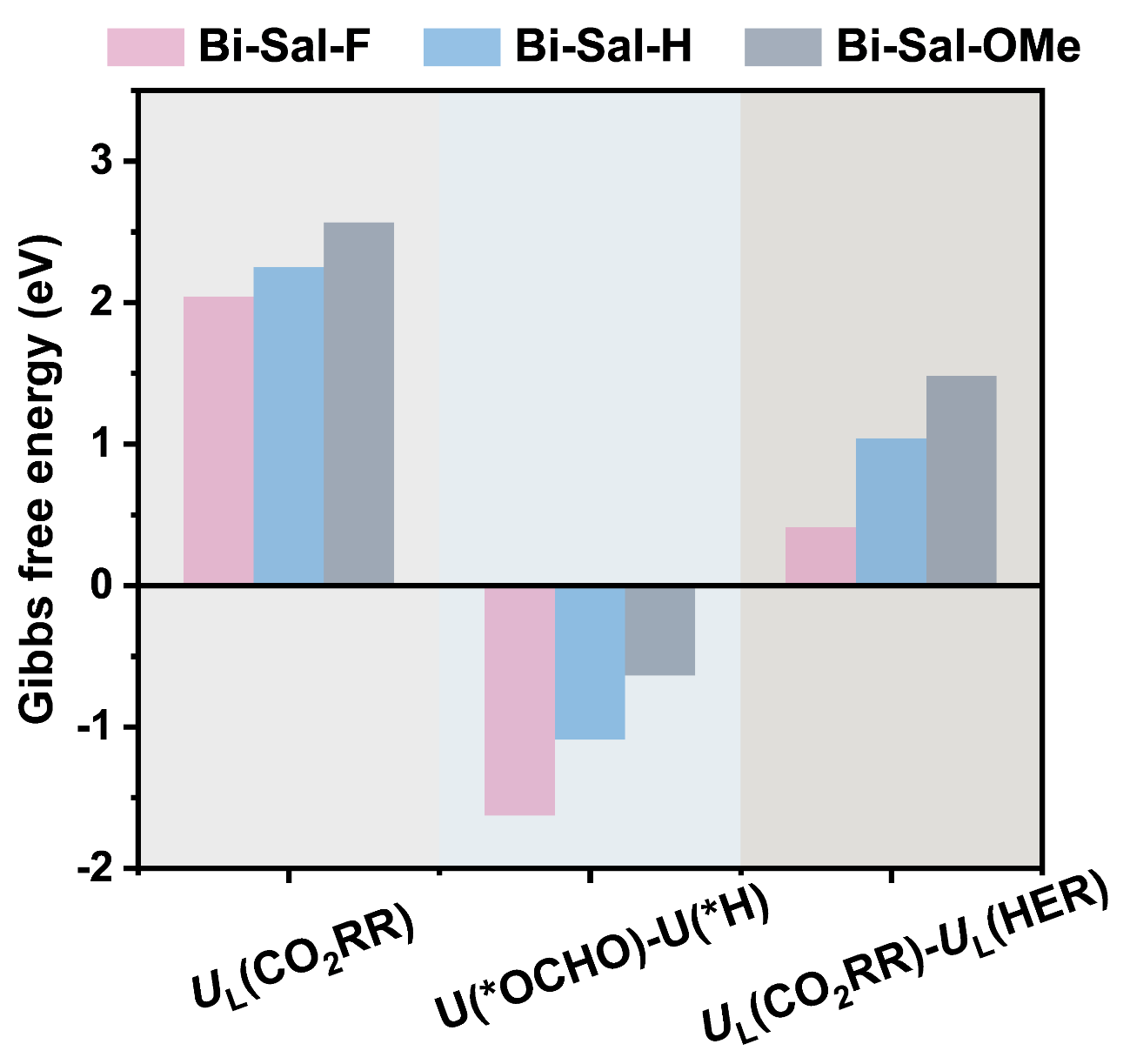
**

**Figure S31.** Comparison of free energy data for CO_2_RR and HER.

*U*_L_(CO_2_RR): the energy barrier for the rate-limiting step of CO_2_RR (*OCHO-to-*HCOOH).

*U*(*OCHO)-*U*(*H): the energy barrier difference between the CO_2_RR intermediate (*OCHO) and the HER intermediate (*H).

*U*_L_(CO_2_RR)- *U*_L_(HER): the difference in energy barriers between the rate-limiting steps of CO_2_RR and HER.


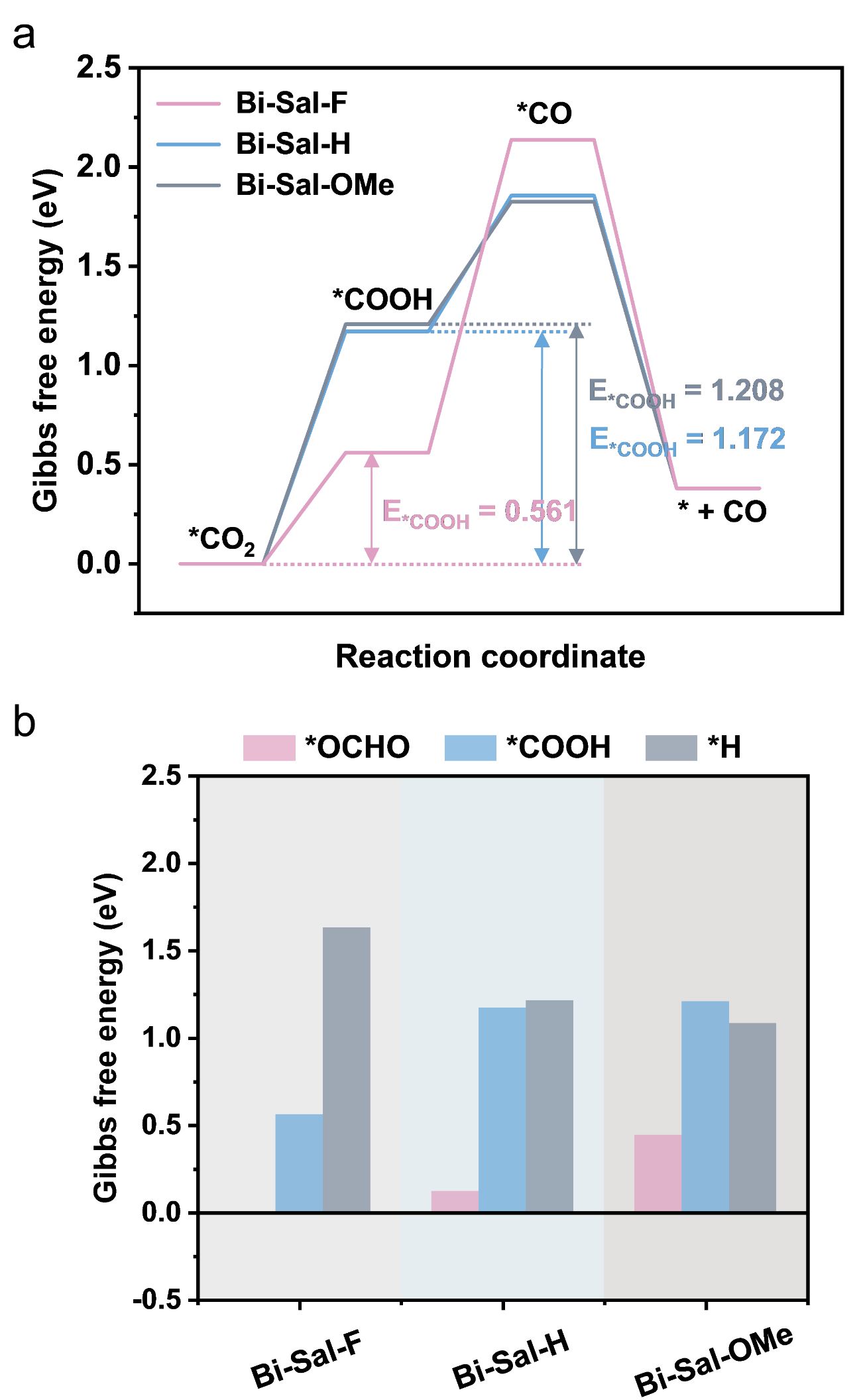


**Figure S32.** (a) Free energy diagrams for the CO_2_RR on Bi-Sal-R, illustrating the CO production via the *COOH pathway. (b) Comparison of free energy data for *OCHO, *COOH, *H on Bi-Sal-R.

**Table S1.** The atomic% ratio of Bi, C, N, O, F in Bi-Sal-R. The result was determined by elemental mapping.

| Sample | Bi at.% | C at.% | N at.% | O at.% | F at.% |
| --- | --- | --- | --- | --- | --- |
| Bi-Sal-F | 1.54 | 81.64 | 5.88 | 6.85 | 4.08 |
| Bi-Sal-H | 1.89 | 81.28 | 6.43 | 10.40 | - |
| Bi-Sal-OMe | 1.93 | 79.46 | 6.36 | 12.25 | - |

**Table S2.** The weight% ratio of Bi, C, N, O, F in Bi-Sal-R. The result was determined by elemental mapping.

| Sample | Bi wt.% | C wt.% | N wt.% | O wt.% | F wt.% |
| --- | --- | --- | --- | --- | --- |
| Bi-Sal-F | 20.51 | 62.35 | 5.24 | 6.97 | 4.93 |
| Bi-Sal-H | 24.26 | 59.98 | 5.53 | 10.23 | - |
| Bi-Sal-OMe | 24.51 | 58.13 | 5.43 | 11.94 | - |

**Table S3.** Element contents measured by ICP-MS.

|  | Bi wt.% by ICP-MS | Theoretical value (%) |
| --- | --- | --- |
| Bi-Sal-F | 30.2 | 37.4 |
| Bi-Sal-H | 34.0 | 39.9 |
| Bi-Sal-OMe | 29.6 | 35.8 |

**Table S4.** Comparison of the Bi 4f of catalysts through experimental data (XPS results).

| Sample | Orbital | Binding energy (eV) | FWHM | Area (%) |
| --- | --- | --- | --- | --- |
| Bi-Sal-F | Bi 4f_7/2_ | 159.82 | 1.37 | 56.5 |
|  | Bi 4f_5/2_ | 165.13 | 1.37 | 43.5 |
| Bi-Sal-H | Bi 4f_7/2_ | 159.68 | 1.33 | 56.4 |
|  | Bi 4f_5/2_ | 164.99 | 1.35 | 43.6 |
| Bi-Sal-OMe | Bi 4f_7/2_ | 159.57 | 1.32 | 55.7 |
|  | Bi 4f_5/2_ | 164.89 | 1.36 | 44.3 |

**Table S5.** Bi *L*_3_-edge EXAFS curve fitting parameters for various samples.

| Sample | Path | CN | R (Å) | σ^2^ (Å^2^) | ΔE_0_ (eV) | R factor |
| --- | --- | --- | --- | --- | --- | --- |
| Bi powder | Bi-Bi1 | 2.8 | 3.10 | 0.007 | 2.60 | 0.019 |
|  | Bi-Bi2 | 3.0 | 3.51 | 0.024 |  |  |
| Bi_2_O_3_ | Bi-O | 2.8 | 2.16 | 0.004 | -2.63 | 0.018 |
|  | Bi-Bi | 4.0 | 3.58 | 0.007 |  |  |
| Bi-Sal-F | Bi-N/O | 4.0 | 2.21 | 0.005 | -4.21 | 0.019 |
|  | Bi-C | 6.3 | 3.21 | 0.006 |  |  |
| Bi-Sal-H | Bi-N/O | 4.2 | 2.20 | 0.006 | -5.56 | 0.016 |
|  | Bi-C | 6.2 | 3.21 | 0.007 |  |  |
| Bi-Sal-OMe | Bi-N/O | 4.3 | 2.23 | 0.009 | -3.43 | 0.019 |
|  | Bi-C | 5.7 | 3.27 | 0.007 |  |  |

^a^ For various samples, S_0_^2^ was fitted as 0.75, according to the experimental EXAFS fit by fixing CN as the known crystallographic value. R is the inter-atomic distance between Bi central atom and the surrounding coordination atom). σ^2^ is Debye-Waller factor. ΔE_0_ is the inner potential correction. R factor indicates the goodness of the fit.

**Table S6.** Parameters obtained by fitting the Nyquist plots of catalysts using the equivalent circuit in Figure S22.

|  | R_s_ / Ω | CPE-T | CPE-P | R_ct_ / Ω |
| --- | --- | --- | --- | --- |
| Bi-Sal-F | 3.19 | 0.0064 | 0.91 | 1.49 |
| Bi-Sal-H | 3.25 | 0.0063 | 0.91 | 3.10 |
| Bi-Sal-OMe | 3.33 | 0.0061 | 0.92 | 5.00 |

**Table S7.** Comparison of the CO_2_RR performance of CO_2_-to-HCOOH catalysts that have been reported in recent years in flow cell, including SACs, metals, and compounds.

| **Sample** | **Electrolyte** | **Potential**  **(V vs RHE)** | ***J*_formate_**  **(mA cm^-2^)** | **FE_formate_ (%)** | **Ref.** |
| --- | --- | --- | --- | --- | --- |
| Bi-Sal-F | 1 M KOH | -1.16 | 471 | 94.1% | **This work** |
| In_2_O_3_@C | 1 M KOH | -1.3 | 138 | ~91% | 15^15^ |
| Bi-TiO_2_-700 | 1 M KOH | -1.0 | 102.6 | 95.8% | 16^16^ |
| SnPc-8F@CNTs | 1 M KOH | -1.2 | 404 | 91.7% | 17^17^ |
| BOC NFs | 1 M KOH | -1.4 | 316 | 97% | 18^18^ |
| Cu_2_SnS_3_ | 1 M KOH | -1.1 | 200.8 | 90% | 19^19^ |
| ZnInS4 | 1 M KOH | -1.2 | 245 | 94% | 20^20^ |
| Bi LNSs | 1 M KOH | -1.1 | 147 | 92% | 21^21^ |
| CT/h-BiOBr | 1 M KOH | -1.3 | 316 | 87% | 22^22^ |
| Bi_2_S_3_-PPy | 1 M KOH | -1.18 | 390 | 90% | 23^23^ |
| Bi NBs | 1 M KOH | -1.47 | 330 | 95% | 24^24^ |
| Ni-In_2_O_3_@C NFs | 1 M KOH | -1.0 | 358 | 90.5% | 25^25^ |
| Bi_2_O_3_@C/HB | 1 M KOH | -1.5 | 285 | 95% | 26^26^ |
| Bi/Bi(Sn)O_x_  NW_s_ | 1 M KOH | -1.0 | 256 | 85% | 27^27^ |
| SnS/GDL | 1 M KOH | -1.3 | ~128 | 88% | 28^28^ |
| SnO_2_/CF | 1 M KOH | -1.2 | 175 | 90% | 29^29^ |
| Bi_2_S_3_-Bi_2_O_3_ NSs | 1 M KOH | -1.0 | ~140 | 95% | 30^30^ |
| SnO_2_-Bi_2_O_3_ | 1.0 M KOH | -1.29 | 220 | 91% | 31^31^ |

**Table S8.** Adsorption free energy was calculated for each step of the CO_2_RR through the *OCHO-to-HCOOH pathway on Bi-Sal-R. Unit: eV

|  | *CO_2_ | *OCHO | *HCOOH | *+HCOOH | rate-limiting step |
| --- | --- | --- | --- | --- | --- |
| Bi-Sal-F | 0 | 0.00084 | 2.041 | 0.34204 | 2.041 |
| Bi-Sal-H | 0 | 0.12064 | 2.37237 | 0.34205 | 2.252 |
| Bi-Sal-OMe | 0 | 0.44265 | 3.00784 | 0.34203 | 2.565 |

**Table S9.** Bond lengths and bending angle of CO_2_ molecules adsorbed on Bi-Sal-R.

|  | Bi-O (Å) | Bi-C (Å) | Bi-O-C (Å) | CO_2_ bending angle |
| --- | --- | --- | --- | --- |
| Bi-Sal-F | 2.50 | 2.828 | 92.4 | 174.5 |
| Bi-Sal-H | 2.61 | 2.634 | 77.98 | 179.0 |
| Bi-Sal-OMe | 2.70 | 2.714 | 77.98 | 192.7 |

**Table S10.** Bond lengths and bond angles of *HCOOH adsorbed on Bi-Sal-R.

|  | BiO1 | BiO2 | Bi-C | Bond angle  (O1-Bi-O2) |
| --- | --- | --- | --- | --- |
| Bi-Sal-F | 2.58 | 2.62 | 2.98 | 50.5 |
| Bi-Sal-H | 2.56 | 2.59 | 2.96 | 51.0 |
| Bi-Sal-OMe | 2.55 | 2.60 | 2.95 | 51.5 |

Bi-O1 and Bi-O2: Distance between Bi and O in HCOOH,

Bi-C: Distance between Bi and C in HCOOH,

O1-Bi-O2: Bond angle formed by Bi with O1 and O2 upon HCOOH adsorption.

**Table S11.** he adsorption free energy for each HER step on Bi-Sal-R. Unit: eV

|  | *H_2_O | | *H | H_2_ | rate-limiting step |
| --- | --- | --- | --- | --- | --- |
| Bi-Sal-F | | 0 | 1.631 | 0 | 1.631 |
| Bi-Sal-H | | 0 | 1.214 | 0 | 1.214 |
| Bi-Sal-OMe | | 0 | 1.084 | 0 | 1.084 |

**Table S12.** The difference in energy barriers between CO_2_RR and HER. Unit: eV

|  | *U*_L_(CO_2_RR) | *U*(*OCHO)-*U*(*H) | *U*_L_(CO_2_RR)-*U*_L_(HER) |
| --- | --- | --- | --- |
| Bi-Sal-F | 2.041 | -1.630 | 0.41 |
| Bi-Sal-H | 2.252 | -1.093 | 1.038 |
| Bi-Sal-OMe | 2.565 | -0.641 | 1.481 |

**Table S13.** Adsorption free energy was calculated for each step of the CO_2_RR through the *COOH-to-CO pathway on Bi-Sal-R. Unit: eV

|  | *CO_2_ | *COOH | *CO | * + CO |
| --- | --- | --- | --- | --- |
| Bi-Sal-F | 0 | 0.561 | 2.137 | 0.380 |
| Bi-Sal-H | 0 | 1.172 | 1.856 | 0.380 |
| Bi-Sal-OMe | 0 | 1.208 | 1.825 | 0.380 |

**References**

1. Xu, W.; Chen, J.; Qiu, Y.; Peng, W.; Shi, N.; Zhou, J., Highly efficient microwave catalytic oxidation degradation of 4-nitrophenol over magnetically separable NiCo_2_O_4_-Bi_2_O_2_CO_3_ composite without adding oxidant. *Separation and Purification Technology* **2019,** *213*, 426-436.

2. Ren, X.; Liu, F.; Wu, H.; Lu, Q.; Zhao, J.; Liu, Y.; Zhang, J.; Mao, J.; Wang, J.; Han, X.; Deng, Y.; Hu, W., Reconstructed Bismuth Oxide through in situ Carbonation by Carbonate-containing Electrolyte for Highly Active Electrocatalytic CO_2_ Reduction to Formate. *Angewandte Chemie International Edition* **2024,** *63* (9), e202316640.

3. Yalçınkaya, B.; Yumbul, E.; Mozioğlu, E.; Akgoz, M., Comparison of DNA extraction methods for meat analysis. *Food chemistry* **2017,** *221*, 1253-1257.

4. Ni, Q.; Zhang, M.; Tang, B.; Hou, W.; Wang, K.; Guo, H.; Zhang, J.; Han, T.; Wu, M.; Wang, L., Rapid synthesis of carbon quantum dot-integrated metal–organic framework nanosheets via electron beam irradiation for selective 5-hydroxymethylfurfural electrooxidation. *Advanced Powder Materials* **2025,** *4* (2), 100267.

5. Hua, J.; Hua, P.; Qin, K., Highly fluorescent N, F co-doped carbon dots with tunable light emission for multicolor bio-labeling and antibacterial applications. *Journal of Hazardous Materials* **2023,** *459*, 132331.

6. Zou, H.; Zhao, G.; Dai, H.; Dong, H.; Luo, W.; Wang, L.; Lu, Z.; Luo, Y.; Zhang, G.; Duan, L., Electronic Perturbation of Copper Single-Atom CO_2_ Reduction Catalysts in a Molecular Way. *Angewandte Chemie International Edition* **2023,** *62* (6), e202217220.

7. Bai, S.; Shao, Q.; Feng, Y.; Bu, L.; Huang, X., Highly Efficient Carbon Dioxide Hydrogenation to Methanol Catalyzed by Zigzag Platinum–Cobalt Nanowires. *Small* **2017,** *13* (22), 1604311.

8. Guo, C.; Gao, Y.; Li, S.-Q.; Wang, Y.; Yang, X.-J.; Zhi, C.; Zhang, H.; Zhu, Y.-F.; Chen, S.; Chou, S.-L.; Dou, S.-X.; Xiao, Y.; Luo, X., Chemical-Stabilized Aldehyde-Tuned Hydrogen-Bonded Organic Frameworks for Long-Cycle and High-Rate Sodium-Ion Organic Batteries. *Advanced Functional Materials* **2024,** *34* (21), 2314851.

9. Dutta, A.; Vasilyev, G.; Vilensky, R.; Zussman, E., Controlling spatiotemporal mechanics of globular protein-polymer hydrogel via metal-coordination interactions. *Chemical Engineering Journal* **2024,** *482*, 148881.

10. Ma, S.; Yang, D.; Guan, Y.; Yang, Y.; Zhu, Y.; Zhang, Y.; Wu, J.; Sheng, L.; Liu, L.; Yao, T., Maximally exploiting active sites on Yolk@shell nanoreactor: Nearly 100% PMS activation efficiency and outstanding performance over full pH range in Fenton-like reaction. *Applied Catalysis B: Environmental* **2022,** *316*, 121594.

11. Dung, N. T.; Thao, V. D.; Thao, N. P.; Thuy, C. T. M.; Nam, N. H.; Ngan, L. V.; Lin, K.-Y. A.; Khiem, T. C.; Huy, N. N., Turning peroxymonosulfate activation into singlet oxygen-dominated pathway for ofloxacin degradation by co-doping N and S into durian peel-derived biochar. *Chemical Engineering Journal* **2024,** *483*, 149099.

12. Su, Y.; Cheng, Y.; Li, Z.; Cui, Y.; Yang, C.; Zhong, Z.; Song, Y.; Wang, G.; Zhuang, L., Exploring the impact of Nafion modifier on electrocatalytic CO_2_ reduction over Cu catalyst. *Journal of Energy Chemistry* **2024,** *88*, 543-551.

13. Wang, M.; Wan, L.; Luo, J., Promoting CO_2_ electroreduction on CuO nanowires with a hydrophobic Nafion overlayer. *Nanoscale* **2021,** *13* (6), 3588-3593.

14. Zhou, L.; Li, C.; Lv, J.-J.; Wang, W.; Zhu, S.; Li, J.; Yuan, Y.; Wang, Z.-J.; Zhang, Q.; Jin, H.; Wang, S., Synergistic regulation of hydrophobicity and basicity for copper hydroxide-derived copper to promote the CO_2_ electroreduction reaction. *Carbon Energy* **2023,** *5* (6), e328.

15. Wang, Z.; Zhou, Y.; Liu, D.; Qi, R.; Xia, C.; Li, M.; You, B.; Xia, B. Y., Carbon-Confined Indium Oxides for Efficient Carbon Dioxide Reduction in a Solid-State Electrolyte Flow Cell. *Angewandte Chemie International Edition* **2022,** *61* (21), e202200552.

16. Jia, G.; Wang, Y.; Sun, M.; Zhang, H.; Li, L.; Shi, Y.; Zhang, L.; Cui, X.; Lo, T. W. B.; Huang, B.; Yu, J. C., Size Effects of Highly Dispersed Bismuth Nanoparticles on Electrocatalytic Reduction of Carbon Dioxide to Formic Acid. *Journal of the American Chemical Society* **2023,** *145* (25), 14133-14142.

17. Chen, B.; Zou, H.; Gong, L.; Zhang, H.; Li, N.; Pan, H.; Wang, K.; Yang, T.; Liu, Y.; Duan, L.; Liu, J.; Jiang, J., Molecular engineering of dispersed tin phthalocyanine on carbon nanotubes for selective CO_2_ reduction to formate. *Applied Catalysis B: Environment and Energy* **2024,** *344*, 123650.

18. Sui, P.-F.; Gao, M.-R.; Liu, S.; Xu, C.; Zhu, M.-N.; Luo, J.-L., Carbon Dioxide Valorization via Formate Electrosynthesis in a Wide Potential Window. *Advanced Functional Materials* **2022,** *32* (32), 2203794.

19. Wang, W.; Wang, Z.; Yang, R.; Duan, J.; Liu, Y.; Nie, A.; Li, H.; Xia, B. Y.; Zhai, T., In Situ Phase Separation into Coupled Interfaces for Promoting CO_2_ Electroreduction to Formate over a Wide Potential Window. *Angewandte Chemie International Edition* **2021,** *60* (42), 22940-22947.

20. Wang, Z.; Qi, R.; Liu, D.; Zhao, X.; Huang, L.; Chen, S.; Chen, Z.; Li, M.; You, B.; Pang, Y.; Yu Xia, B., Exfoliated Ultrathin ZnIn_2_S_4_ Nanosheets with Abundant Zinc Vacancies for Enhanced CO_2_ Electroreduction to Formate. *ChemSusChem* **2021,** *14* (3), 852-859.

21. Wang, D.; Liu, C.; Zhang, Y.; Wang, Y.; Wang, Z.; Ding, D.; Cui, Y.; Zhu, X.; Pan, C.; Lou, Y.; Li, F.; Zhu, Y.; Zhang, Y., CO_2_ Electroreduction to Formate at a Partial Current Density up to 590 mA mg^−1^ via Micrometer-Scale Lateral Structuring of Bismuth Nanosheets. *Small* **2021,** *17* (29), 2100602.

22. Chen, Y.; Zhang, Y.; Li, Z.; Liu, M.; Wu, Q.; Lo, T. W. B.; Hu, Z.; Lee, L. Y. S., Amphipathic Surfactant on Reconstructed Bismuth Enables Industrial-Level Electroreduction of CO_2_ to Formate. *ACS Nano* **2024,** *18* (29), 19345-19353.

23. Li, C.; Liu, Z.; Zhou, X.; Zhang, L.; Fu, Z.; Wu, Y.; Lv, X.; Zheng, G.; Chen, H., Bio-inspired engineering of Bi_2_S_3_–PPy composite for the efficient electrocatalytic reduction of carbon dioxide. *Energy & Environmental Science* **2023,** *16* (9), 3885-3898.

24. Zeng, G.; He, Y.; Ma, D.-D.; Luo, S.; Zhou, S.; Cao, C.; Li, X.; Wu, X.-T.; Liao, H.-G.; Zhu, Q.-L., Reconstruction of Ultrahigh-Aspect-Ratio Crystalline Bismuth–Organic Hybrid Nanobelts for Selective Electrocatalytic CO_2_ Reduction to Formate. *Advanced Functional Materials* **2022,** *32* (30), 2201125.

25. Chen, Z.; Yu, G.; Li, B.; Zhang, X.; Jiao, M.; Wang, N.; Zhang, X.; Liu, L., In Situ Carbon Encapsulation Confined Nickel-Doped Indium Oxide Nanocrystals for Boosting CO_2_ Electroreduction to the Industrial Level. *ACS Catalysis* **2021,** *11* (23), 14596-14604.

26. Liu, S.-Q.; Shahini, E.; Gao, M.-R.; Gong, L.; Sui, P.-F.; Tang, T.; Zeng, H.; Luo, J.-L., Bi_2_O_3_ Nanosheets Grown on Carbon Nanofiber with Inherent Hydrophobicity for High-Performance CO_2_ Electroreduction in a Wide Potential Window. *ACS Nano* **2021,** *15* (11), 17757-17768.

27. Zhao, Y.; Liu, X.; Liu, Z.; Lin, X.; Lan, J.; Zhang, Y.; Lu, Y.-R.; Peng, M.; Chan, T.-S.; Tan, Y., Spontaneously Sn-Doped Bi/BiO_x_ Core–Shell Nanowires Toward High-Performance CO_2_ Electroreduction to Liquid Fuel. *Nano Letters* **2021,** *21* (16), 6907-6913.

28. Zou, J.; Lee, C.-Y.; Wallace, G. G., Boosting Formate Production from CO_2_ at High Current Densities Over a Wide Electrochemical Potential Window on a SnS Catalyst. *Advanced Science* **2021,** *8* (15), 2004521.

29. Ning, S.; Wang, J.; Xiang, D.; Huang, S.; Chen, W.; Chen, S.; Kang, X., Electrochemical reduction of SnO_2_ to Sn from the Bottom: In-Situ formation of SnO_2_/Sn heterostructure for highly efficient electrochemical reduction of carbon dioxide to formate. *Journal of Catalysis* **2021,** *399*, 67-74.

30. Sui, P.-F.; Xu, C.; Zhu, M.-N.; Liu, S.; Liu, Q.; Luo, J.-L., Interface-Induced Electrocatalytic Enhancement of CO_2_-to-Formate Conversion on Heterostructured Bismuth-Based Catalysts. *Small* **2022,** *18* (1), 2105682.

31. Wang, X.; Wang, W.; Zhang, J.; Wang, H.; Yang, Z.; Ning, H.; Zhu, J.; Zhang, Y.; Guan, L.; Teng, X.; Zhao, Q.; Wu, M., Carbon sustained SnO_2_-Bi_2_O_3_ hollow nanofibers as Janus catalyst for high-efficiency CO_2_ electroreduction. *Chemical Engineering Journal* **2021,** *426*, 131867.
